# Towards an understanding of retouch flakes: A use-wear blind test on knapped stone microdebitage

**DOI:** 10.1371/journal.pone.0243101

**Published:** 2020-12-07

**Authors:** Benjamin Chan, Juan Francisco Gibaja, Virginia García-Díaz, Christian Steven Hoggard, Niccolò Mazzucco, Jake Thomas Rowland, Annelou Van Gijn

**Affiliations:** 1 Archaeology, Faculty of Arts and Humanities, University of Southampton, Southampton, United Kingdom; 2 Laboratory for Material Culture Studies, Faculty of Archaeology, Leiden University, Leiden, The Netherlands; 3 Institución Milá y Fontanals (IMF-CSIC), Department of Archaeology and Anthropology, Barcelona, Spain; Ghent University, BELGIUM

## Abstract

The retouching and resharpening of lithic tools during their production and maintenance leads to the production of large numbers of small flakes and chips known as microdebitage. Standard analytical approaches to this material involves the mapping of microartefact densities to identify activity areas, and the creation of techno-typologies to characterise the form of retouch flakes from different types of tools. Whilst use-wear analysis is a common approach to the analysis of tools, it has been applied much less commonly to microdebitage. This paper contends that the use-wear analysis of microdebitage holds great potential for identifying activity areas on archaeological sites, representing a relatively unexplored analytical resource within microartefact assemblages. In order to test the range of factors that affect the identification of use-wear traces on small retouch flakes, a blind test consisting of 40 retouch flakes was conducted. The results show that wear traces can be identified with comparable levels of accuracy to those reported for historic blind tests of standard lithic tools suggesting that the use-wear analysis of retouch flakes can be a useful analytical tool in understanding site function, and in increasing sample sizes in cases where assemblages contain few tools.

## Introduction

Blind tests have been a central component in the development of use-wear studies for forty years [e.g. 1–6]. To date, these blind tests have been conducted on a variety of both knapped and occasionally ground stone tools [7], and have been designed to address a range of research questions. One feature these blind tests have in common is that they have involved the analysis of complete or substantially complete tools. This is a reflection of archaeological use-wear applications, which are also predominantly focused on the analysis of tools. Much less rarely subjected to use-wear analysis are the small flakes that are either detached from tools unintentionally during their use, or intentionally during their retouching or resharpening. The analysis of these small flakes, referred to as microdebitage, retouch flakes, or resharpening flakes, fall within the broader field of microartefact studies. Use-wear analysis has occasionally been conducted on retouch flakes [8], and the suitability of the material for use-wear analysis may seem self-evidential. However, retouch flakes can measure as little as 1mm across, and there has been no study of the effects that their size has on the accuracy with which wear traces can be identified on them. Standard use-wear approaches involve the mapping of wear traces—edge rounding, striations, edge removals, and polish—across the surface of tools [9, 10]. Whilst use-wear analysis is predominantly microscopic in its approach, the analysis of the distribution of wear traces is heavily macroscopic in nature. Often ephemeral traces need to be understood in relation to each other, and to an object’s morphology, to arrive at a reasoned understanding of the use of a tool, a process that is both nuanced and necessarily interpretative in its approach [11]. Generating an understanding of a tool’s use through the systematic mapping of traces across its surfaces is not possible in the case of retouch flakes, which represent only a small fragment of the edge of the parent-tool. Some detailed consideration of the effects on functional analysis of the removal of retouch flakes from the overall context of the tool is therefore warranted if we are to understand the potentials of the use-wear analysis of this material. In line with the development of the discipline as a whole, the appropriate method for establishing this is to conduct a blind test designed to test the parameters that affect the identification of wear traces on retouch flakes. The blind test involved the creation of an assemblage of 40 retouch flakes which were subject to a blind test by three different testers.

## Research context

### Microecofact, microartefact and microdebitage studies

The study of microremains (i.e. the study of microartefacts and microecofacts) form part of a suite of approaches, also including micromorphological and geochemical analysis of soils, aimed at investigating the use of space, and particularly at identifying activity areas, across sites [12]. The purpose of these analyses is most often to better understand site function, variability within households/domestic units, or the organisation of technological practices across sites. This information can enhance our knowledge of social differentiation, context and mode of skill acquisition, and the social role of subsistence and craft activities within past societies [13–17]. The basic principle of the study of microremains is that macroartefacts and macroecofacts are often removed from their original location of use, whilst microartefacts and microecofacts are more likely to get trodden or swept into house floors and other occupation surfaces. Therefore, when compared to macroartefact distributions, the distribution of microartefacts is more likely to reflect the locations of activity areas [18–21]. It should be made clear that despite the sound basis behind their study, it would be wrong to simply accept the densities of microremains as being representative of the locations of activity areas. This has been demonstrated clearly at Çatalhöyük where the houses were frequently replastered and analysis has shown that densities of microartefacts within wall plasters are as high as those within floor plasters, indicating that much of the material was incorporated unintentionally during the mixing of plaster, rather than the use of the floor [12, 15, 22]. Therefore, site formation processes need to be considered on a site-by-site basis before microartefact distribution patterns can be interpreted.

The range of artefacts and ecofacts that the analysis of microremains has been applied to is diverse, with analysis of the distributions of carbonised plant remains, bone, pottery and lithic microartefacts being the most common. The components of bone and pottery microartefact and microecofact assemblages generally represent material that has become fragmented through a variety of agencies and taphonomic processes. The heavily fragmented character of these assemblages is perhaps the principal reason why their study most commonly involves analysis of the spatial distribution of their density without any attempt to sub-categorise or otherwise analyse the material. In contrast, the components of chipped stone microartefact assemblages are not comprised of fragmentary objects, rather their most common constituent is microdebitage. The size definition of microdebitage varies between authors from <1mm to <20mm [23, 24], however, as a term it relates to small debitage (i.e. flakes) produced either unintentionally as a by-product of knapping or tool use, or intentionally during the trimming and faceting of core platforms and the retouching/resharpening of the edges of tools. The latter involves the removals of *retouch flakes*, which are defined as flakes removed intentionally to modify the edge of a tool during manufacture, re-sharpening, or reworking. *Resharpening flakes* are defined as flakes removed intentionally from the edge of a used tool in order to resharpen its edge. *Reworking* is defined as the reshaping of a tool in order to substantially alter the morphology of an edge, or repurpose it to a different use. Whilst lithic microdebitage has been included in standard density based microartefact analyses, its greater analytical potential has also been explored in specialist analyses focused on the identification of retouch flakes.

The first detailed techno-typological analysis of chipped stone microartefacts was undertaken by Frison [25] at Piney Creek, Wyoming. Through refitting, Frison showed that the re-sharpening of scrapers and bifaces produced technologically distinctive flakes which could be identified archaeologically. Schiffer [26] and Binford [27] also recognised that the presence of microdebitage flakes was more likely to indicate the location of manufacture or retouch events than the presence of the tools themselves. This was supported by later research correlating the spatial distribution of microdebitage scatters with truly microscopic debitage flakes (<1mm) in sediments [23, 28, 29]. This demonstrated that the microdebitage flakes had not moved significantly post-depositionally and that their presence indicated *in situ* knapping events.

Complementing Frison’s [25] earlier work, Newcomer & Karlin’s [30] work at the late Magdalenian site of Pincevent, France highlighted the interpretive potential of microdebitage beyond locating *in situ* knapping activities. Their research demonstrated that a microdebitage typology based on diagnostic technological attributes could be used to identify the manufacture of different tool types at sites. Adopting this approach Nadel [31] developed a typology of microdebitage produced from blade core reduction at the site of Ohalo II, Jordan. Spatial mapping of these types across the site enabled the locations of core reduction activities to be identified.

Moving beyond techno-typological classification systems, the application of functional use-wear analyses of microdebitage has been rare but has in some cases proved instrumental in interpreting site function. Working with lithic assemblages from rock shelter sites from Australia and East Timor dated from 40,000–50,000 BP Hayes *et al*. [8] identified that a high proportion of use-wear traces were located solely on the external platform edge of small flakes. They deduced that these artefacts must represent either retouch flakes, or flakes detached during use, and suggested that these flakes were a better indicator of site function than retouched tools, which were often moved around from one site to another. At the Federmesser site of Rekem, Belgium De Bie *et al*. [32] conducted extensive refitting, spatial analysis and use-wear analysis on the clustered artefact scatters from the site. Their findings included the identification of wear traces on burin spalls, revealing not only the locations of particular activities, but also providing insight into the cycle of production, use, resharpening, and reuse of individual tools.

Despite the potential highlighted by these cases, use-wear analysis of retouch flakes and microdebitage has seen limited application. This paper contends that a combined functional and techno-typological analysis of lithic microdebitage provides great analytical potential in comparison to other forms of microartefact analyses. The analysis has the potential to not only define the location of activity areas, but also the types of tools that were being used, the types of technological activities being practiced (tool production vs. tool maintenance), and the types of material that tools were being used upon.

Beyond the identification of activity areas, the use-wear analysis of microdebitage also has the potential to increase sample sizes within contexts and sites where there are few tools present. Gaining meaningful interpretation on the basis of relatively small samples of objects with identifiable wear traces is a relatively common problem faced by use-wear analysts. Given that the material in question here usually remains unstudied by use-wear analysts, any site with a reasonable assemblage of microdebitage has the potential to resolve this issue.

In light of the above, the main aim of the blind test was to investigate the range of factors affecting the identification of use-wear traces on retouch flakes. The initial intention was to assess the extent to which it was possible to distinguish between retouch flakes removed from used tool edges (i.e. resharpening flakes), from those removed from unused edges. Following on from this, if resharpening flakes could be identified, the goal was to test the level of accuracy with which the contact material and directionality of use of the tool could be determined.

## Experiment methodology

Prior to the analysis, the main parameters that were thought to influence the identification of wear traces on retouch flakes were the morphology of the original retouched edge, the size and morphology of the resharpening flake after its removal, the type of hammer used to retouch the edge (soft vs hard), the type of contact material that the edge was used on, the directionality with which the edge was used, and the duration of use of the edge.

In order to focus on the parameters that were of most interest archaeologically the flakes used in the test were all removed from experimental tools that had been used for a standard duration, and that had a standardised edge morphology. Therefore, the key variables that were being assessed in the blind test were the directionality of use, the type of contact material the edge was used on, and the type of hammer used to remove the flake. The reason that the latter was tested was because it is known that the traces left from hammer strikes during knapping have the potential to be misinterpreted as use-related traces on complete tools where entire use-edges are available for analysis [33, 34 p41-42]. With retouch flakes this problem could be exacerbated due to the small size of area available for analysis making it difficult to distinguish between relatively isolated wear traces from hammer strikes and the more extensive traces normally associated with tool-use. In order to assess this potential issue both antler and stone hammers were used to generate retouch flakes.

**Fig 1:**
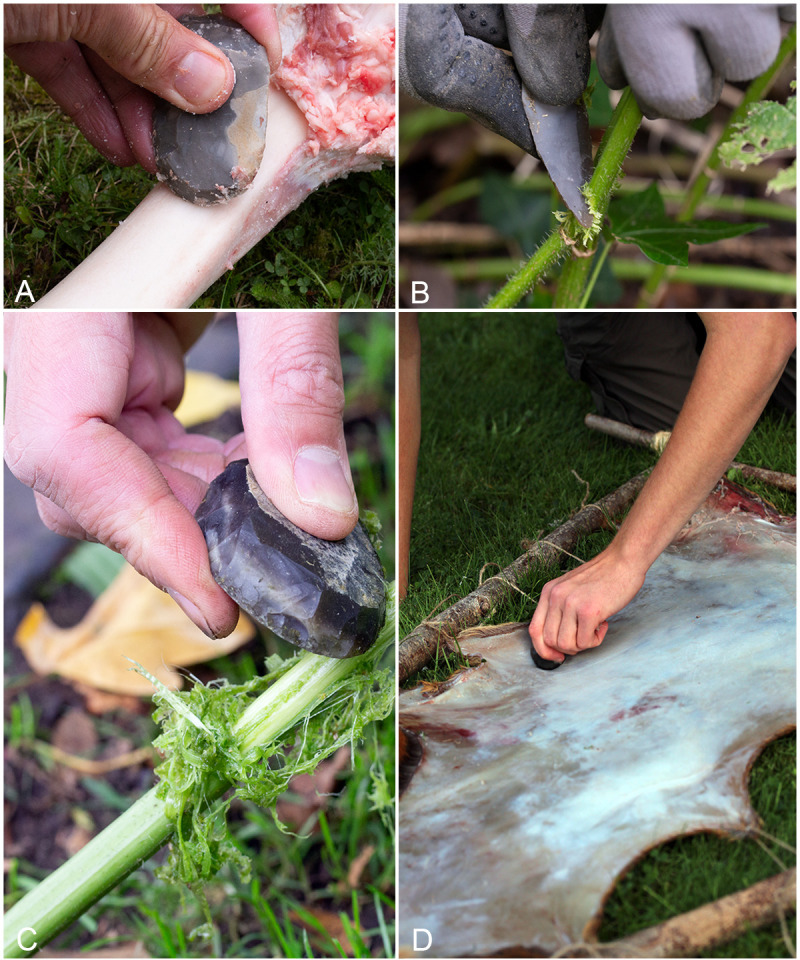
Experimental tools used to produce retouch flakes. A) Tool 14 used to scrape a cattle femur, B) Tool 7 used to harvest nettles, C) Tool 9 used to scrape fibres from nettle stems, D) Tool 4 used to scrape fresh hide.

In terms of edge morphology, tools were used that were made on thick blanks with steep-angled retouched edges, the edge typically found on scrapers. Focusing on retouch flakes from scrapers is particularly useful as they are morphologically distinctive [25, 30], and scrapers are also a near ubiquitous presence within knapped stone assemblages from the Lower Palaeolithic onwards. The tools were used on a range of materials that are commonly identified in the analysis of prehistoric assemblages, and that ranged in hardness from soft to hard (Table 1; Fig 1).

**Table 1:** The tool type, contact material, directionality, type of preparation, retouching hammer type and duration of the experiments used to produce resharpening flakes.

Experiment/ Tool Number	Tool Type	Contact Material	Preparation	Directionality	Retouching Hammer Type	Duration (minutes)
1	Scraper	Dry Hide—Roe deer (*Capreolus capreolus*)	Dried for two weeks	Transverse	Stone	90
2	Scraper	Dry Hide- Roe deer (*Capreolus capreolus*)	Dried for two weeks	Transverse	Antler	90
3	Scraper	Fresh Hide—Roe deer (*Capreolus capreolus*)	No preparation	Transverse	Antler	90
4	Scraper	Fresh Hide—Roe deer (*Capreolus capreolus*)	No preparation	Transverse	Stone	90
5	Scraper	Fresh Bone–Cattle femur (*Bos Taurus*)	No preparation	Transverse	Stone	90
6	Scraper	Fresh Bone–Cattle femur (*Bos Taurus*)	No preparation	Longitudinal	Stone	90
7	Knife	Plant stems–Nettle (*Urtica dioica*)	No preparation	Longitudinal	Antler	90
8	Scraper	Plant stems–Nettle (*Urtica dioica*)	No preparation	Longitudinal	Stone	120
9	Scraper	Plant stems–Nettle (*Urtica dioica*)	No preparation	Transverse	Stone	90
10	Scraper	Plant stems–Nettle (*Urtica dioica*)	No preparation	Transverse	Stone	90
11	Scraper	Green wood/bark—Hazel (*Corylus avellana)*	No preparation	Transverse	Stone	90
12	Scraper	Green wood/bark—Hazel (*Corylus avellana)*	No preparation	Transverse	Stone	90
13	Scraper	Dried Bark–Lime (*Tilia* x *europaea)*	Dried for three months	Transverse	Stone	90
14	Scraper	Fresh Bone–Cattle femur (*Bos Taurus*)	No preparation	Transverse	Stone	90
15	Scraper	Dried Bark–Lime (*Tilia* x *europaea)*	Dried for three months	Transverse	Stone	165
16	Scraper	Nettle Stems–(*Urtica dioica*)	No preparation	Transverse	Antler	150
17	Scraper	Dry Bone–Cattle femur (*Bos Taurus*)	Dried for six months	Longitudinal	Stone	90
18	Scraper	Seasoned wood–Pine (*Pinus sylvestris)*	Seasoned for 12 months	Transverse	Stone	180

The experiments involved the use of commercially available animal products (cattle bone, and deer hide) procured from registered butchers. The roe deer hides used in Experiments 1–4 were purchased from Hampshire Game Ltd. (located at 51°15'26.3"N 1°32'12.5"W) and were from deer carcasses that had been recently skinned. The cattle bones used in Experiments 5, 6, 14 and 17 were purchased in a freshly butchered state from Uptons of Bassett (located at 50°56'03.7"N 1°25'06.4"W). The study also involved the use of collected plant materials. All plant materials are common species in the United Kingdom and were collected from a private garden. No permit was required for their collection. The experiment methodology was presented to the Ethics Committee of the Faculty of Archaeology, Leiden University who granted a formal waiver.

The tools used in the experiments were made by JTR, and used by JTR and BC, both of whom are experienced in using stone tools. Tools were used only for tasks for which they were practically suited to, with the range of experiments also being designed to generate traces from a variety of contact materials and to include longitudinal and transverse directionality. The fact that scrapers are more typically suited to use in a transverse motion, meant that the longitudinal uses were more limited in number. In the case of Experiment 7, a decision was made to make a knife to facilitate the use of a tool in the harvesting of plants (Fig 1). Whilst this tool was morphologically knife-shaped, i.e. an elongated flake with retouched converging lateral margins, the retouched edge was steep angled and it would be difficult morphologically to separate individual retouch flakes from the edge of this tool from those produced from a scraper.

All experiments were conducted for 90 minutes, however, prior to resharpening, it was realised that some of the experiments had only produced weak traces that would have been difficult to identify on complete tools, let alone on the resharpening flakes from those tools. Therefore, these experiments were conducted for a longer period of time until wear traces were more developed (Table 1).

**Fig 2:**
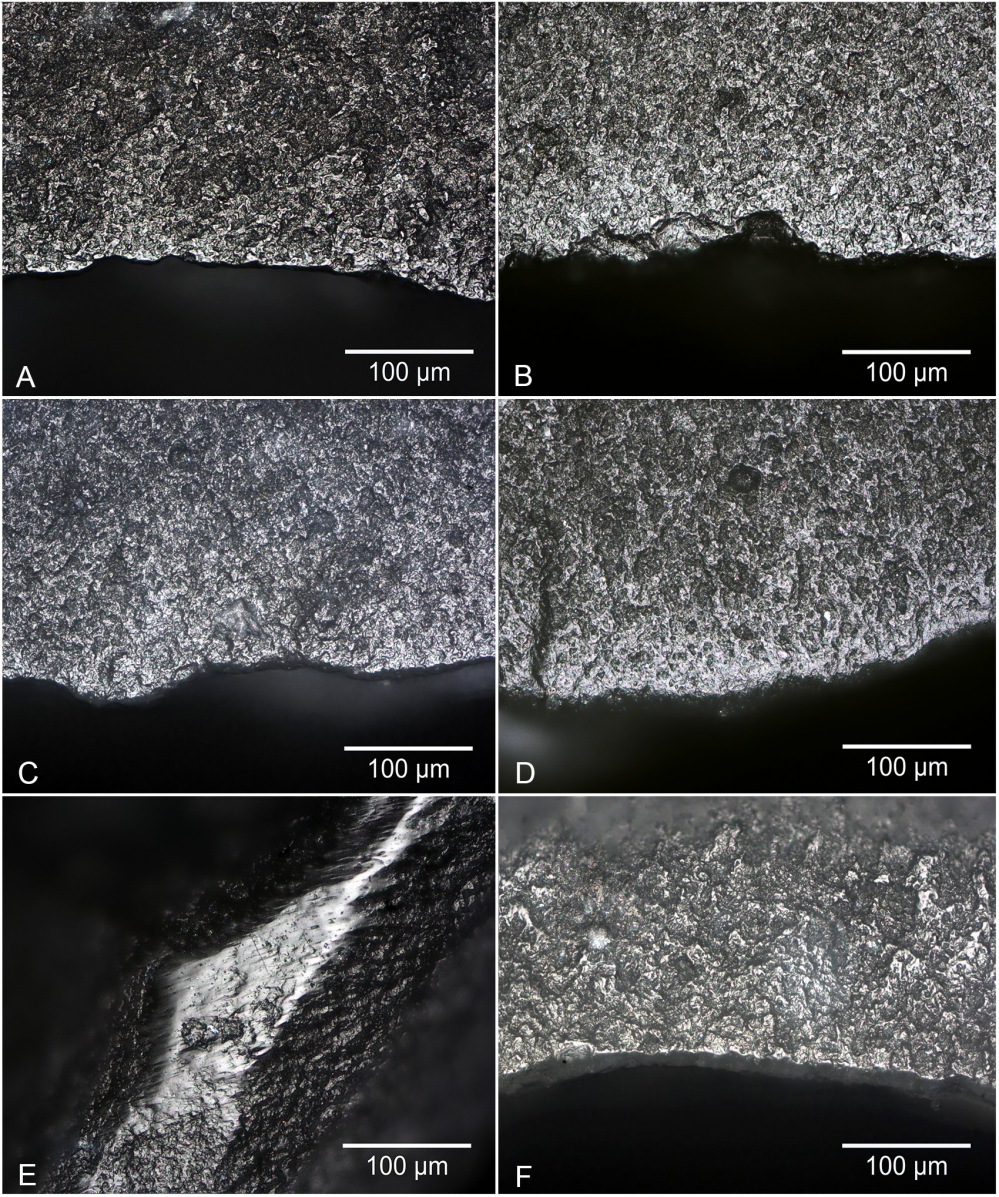
Wear traces on the blind test retouch flakes. A) Flake 23 from a tool used to harvest nettle, B) Flake 20 from a tool used to scrape hazel bark and wood, C) Flake 32 from a tool used to scrape lime bark, D) Flake 2 from a tool used to scrape fresh hide, E) Flake 17 from a tool used to groove bone, F) Flake 21 from a tool used to scrape pine. All images at 200x. Photos A, B, C, D and F show the external platform edge of the butts of retouch flakes, the surface that was originally the ventral side of the use-edge of the parent tool. Photo E shows wear on a dorsal ridge of the retouch flake, which was originally the ridge between two retouch facets on the retouched dorsal side of the use-edge of the parent tool.

After the experiments were completed the traces on the tools were recorded using incident light microscopy and then retouched to produce an assemblage of resharpening flakes (Fig 2). Flakes were also removed from unused tools to produce a control sample.

### Cleaning protocol

In order to fully remove the residues adhering to the surface of the tools they were cleaned with detergent and water, and then placed in an ultrasonic tank with distilled water, HCl 10%, and KOH 10%, each on a 15 minute rotation, followed by an hour in distilled water. Tools were thoroughly rinsed in fresh water between each stage to remove any HCl or KOH still adhering to the tool surface. After the retouching, the retouch flakes were further cleaned using the same procedure in order to remove any residues from the retouching. The retouch flakes were then scanned for signs of remaining residues to ensure that they were fully cleaned before the blind test took place.

### Selection protocol

After the retouch flakes had been cleaned they were scanned for surviving wear traces. A sample was then chosen from the retouch flakes with wear traces covering the full range of contact materials, hammer types and directionality of use represented in the experiments. The sample also incorporated retouch flakes taken from unused tools, as well as some flakes taken from the unused portions of the edges of used tools. The resultant blind test assemblage consisted of 40 flakes varying in weight from 0.0002g to 0.7236g (Table 2). These flakes comprised 32 from used tools with wear traces identified during the initial assessment, three flakes from used tools that had hammer marks but no wear traces identified on them, and five flakes struck from unused tools. Three of the flakes had lost their butts during retouching, but had surviving traces on their dorsal ridges, the rest were complete.

**Table 2:** The sample of retouch flakes used for the blind test (species names of contact materials are provided in Table 1).

Flake Number	Parent tool number	Contact Material	Directionality	Retouch Hammer	Weight of flake (g)	Length (mm)	(Breadth mm)	Note
1	2	Dry Hide	Transverse	Antler	0.0186	4.1	6.2	
2	4	Fresh Hide	Transverse	Stone	0.0503	6.4	8.5	
3	8	Nettle Stems	Longitudinal	Stone	0.0505	3.9	8.5	
4	14	Fresh Bone	Transverse	Stone	0.5263	12.7	14.3	
5	17	Bone	Longitudinal	Stone	0.1807	12.6	9.3	
6	18	Pine	Transverse	Stone	0.0502	6.7	8.6	
7	3	Fresh Hide	Transverse	Antler	0.0018	2.7	2.4	
8	15	Lime Bark	Transverse	Stone	0.0797	10.5	7.6	
9	17	Bone	Longitudinal	Stone	0.2738	9.3	11.3	
10	13	Dried Lime Bark	Transverse	Stone	0.0441	6.4	6.1	
11	14	Fresh Bone	Transverse	Stone	0.1197	8.3	9.4	
12	2	Dry Hide	Transverse	Antler	0.0256	5.6	5.2	
13	6	Fresh Bone	Longitudinal	Stone	0.0656	7.5	6.6	Missing butt but traces on dorsal ridges
14	18	Pine	Transverse	Stone	0.1067	6.8	8.3	
15	17	Bone	Longitudinal	Stone	0.0283	7.2	3.7	Missing butt but traces on dorsal ridges
16	8	Nettle Stems	Longitudinal	Stone	0.0184	4.1	6.5	
17	17	Bone	Longitudinal	Stone	0.0176	4.1	4.5	Missing butt but traces on dorsal ridges
18	2	Dry Hide	Transverse	Antler	0.0002	3.9	3.6	
19	14	Fresh Bone	Transverse	Stone	0.0434	9.7	4.2	Used but no wear traces
20	12	Hazel bark/wood	Transverse	Stone	0.4435	9.8	15.0	
21	18	Pine	Transverse	Stone	0.0640	6.8	9.8	
22	14	Fresh Bone	Transverse	Stone	0.2225	12.0	9.6	
23	7	Nettle Stems	Longitudinal	Antler	0.0999	7.7	9.8	
24	13	Dried Lime Bark	Transverse	Stone	0.0470	7.0	6.2	Used but no wear traces
25	N/A	Unused	Unused	Antler	0.0590	6.3	8.3	From unused tool
26	18	Pine	Transverse	Stone	0.0134	3.5	5.3	
27	12	Hazel bark/wood	Transverse	Stone	0.0986	7.5	9.8	
28	15	Lime Bark	Transverse	Stone	0.2311	12.2	12.7	
29	18	Pine	Transverse	Stone	0.7236	13.7	13.9	
30	4	Fresh Hide	Transverse	Stone	0.0479	5.7	7.6	
31	N/A	Unused	Unused	Stone	0.0521	11.0	5.9	From unused tool
32	15	Lime Bark	Transverse	Stone	0.1949	11.0	9.4	
33	N/A	Unused	Unused	Stone	0.6812	15.0	13.7	From unused tool
34	12	Hazel bark/wood	Transverse	Stone	0.1385	7.3	9.9	
35	N/A	Unused	Unused	Antler	0.0224	4.9	6.2	From unused tool
36	5	Fresh Bone	Transverse	Stone	0.0898	7.1	9.9	
37	16	Nettle Stems	Transverse	Antler	0.0662	5.1	10.5	
38	7	Nettle Stems	Longitudinal	Antler	0.0092	4.4	3.8	
39	N/A	Unused	Unused	Antler	0.1530	10.1	11.2	From unused tool
40	7	Nettle Stems	Longitudinal	Antler	0.0085	3.9	4.0	Used but no wear traces

During the course of the test one of the retouch flakes, Flake 7, was lost. This flake was therefore not available for two of the blind testers to analyse. In order to minimise impact on the results, the flake was replaced with another resharpening flake taken from the same tool, however, Blind Tester 2 was not able to view this flake. This means that two testers looked at 40 flakes, and one tester looked at 39 flakes, making a total of 119 individual responses to the blind test.

### Blind test analysis protocol

The test was taken by three individuals, all of whom were experienced use-wear analysts. The blind testers were not involved in the original experiments, the use-wear analysis of the complete tools, the retouching of the tools to produce the retouch flakes, or the sampling of the retouch flakes to produce the blind test assemblage. The blind testers had no knowledge of the range of contact materials involved in the experiments prior to taking the blind test.

In order to standardise the responses to the test, the blind testers were asked to address a set series of questions. These were as follows:

1. Was the retouch flake was from a used or unused edge?
2. What was the directionality of use?
3. What was the general category of contact material (e.g. soft, hard etc.)?
4. What was the specific category of contact material?
5. What was the location of identified traces?
6. What degree of certainty was there in interpretation?

To maximise comparability in results between testers the resultant variables had defined states, apart from the category for the specific category of contact material, as the potential range of answers needed to be kept broad (Table 3).

**Table 3:** The attributes and attribute states the blind testers were asked to record on the sample of retouch flakes.

**Used/Unused**- Please state whether you think the flake is from a tool that was: 1. Used 2. Unused 3. Indeterminate **Directionality**: Please state what you think the direction of use of the tool was: 1. Transverse (i.e. 90^o^ to the edge of the tool) 2. Longitudinal (i.e. parallel to the edge of tool) 3. None (i.e. unused tool) 4. Indeterminate (i.e. tool used but directionality not apparent) 5. Other (please define) **Contact Material General**- Please state whether you think the contact material was: 1) Soft 2) Medium 3) Hard 4) Indeterminate (i.e. tool used but unclear whether contact material was hard or soft) 5) None (i.e. tool unused) **Contact Material Specific**- Please state as specifically as possible what you think the contact material was (i.e. wood, bone, antler, mineral etc.). If possible, state species or sub-groups within broader categories (i.e. if wood or plant, do you know what species it is? If not, can you refine the general category -i.e. siliceous plants, hard wood etc.) **Location of Identified Traces–**Please state where on the microdebitage you spotted the use-wear traces 1. Butt 2. Dorsal surface 3. Butt and Dorsal Surface 4. No identified traces **Level of certainty in interpretation–**Please state how certain you were in your overall interpretation of the object 1. Uncertain 2. Certain 3. Very Certain **Supporting Notes** Please note anything you want to add about the analysis of the flake. Were the traces easy to find? If you have doubts about your identification, what are they?

Due to the logistics of organising a blind test involving three different researchers based in different countries, each blind tester undertook the test in their own lab using their own microscopes. All testers used both stereoscope (Leica MZ16A), and incident light (Nikon N300 Labophot and Olympus BH2) microscopy.

Blind testers were encouraged to record why they came to certain conclusions about individual flakes, and to take micrographs to illustrate any queries or uncertainties that they had, but otherwise were given no direction in terms of how to analyse the objects.

### Scoring responses and data analysis methodology

The responses to each attribute for each flake were scored as being correct, incorrect, partially correct, or indeterminate (Tables 4–6). Assigning answers to these categories required a degree of latitude in the case of the specific contact material attribute as attribute states could not be pre-defined and multiple responses could be considered to be correct. In these cases the testers were given the opportunity to respond if they disagreed with the way in which their answer had been scored.

**Table 4:** The responses and correctness of responses of Blind Tester 1.

Flake Number	Used/Unused		Directionality		Contact material-General		Contact material—Specific		Location of traces	Level of certainty
	Blind Tester Response	Correctness of response	Blind Tester Response	Correctness of response	Blind Tester Response	Correctness of response	Blind Tester Response	Correctness of response		
1	Used	Correct	Transverse	Correct	Soft	Correct	Soft animal material	Correct	Dorsal surface	Uncertain
2	Used	Correct	Transverse	Correct	Soft/Medium	Correct	Abrasive animal material (Hide)	Correct	Butt and dorsal surface	Very certain
3	Used	Correct	Longitudinal	Correct	Medium/Hard	Incorrect	Ligneous vegetal material (soft wood or reeds)	Partially correct	Butt and dorsal surface	Quite certain
4	Used	Correct	Transverse	Correct	Hard	Correct	Indeterminate	Partially correct	Butt and dorsal surface	Uncertain
5	Used	Correct	Longitudinal	Correct	Hard	Correct	Bone/antler	Correct	Butt and dorsal surface	Very certain
6	Used	Correct	Transverse	Correct	Medium	Correct	Ligneous vegetal material	Correct	Butt and dorsal surface	Very certain
7	Used	Correct	Transverse	Correct	Medium	Partially correct	Abrasive animal material (Hide)	Correct	Dorsal surface	Very certain
8	Used	Correct	Transverse	Correct	Medium/Hard	Correct	Vegetal material	Correct	Butt and dorsal surface	Uncertain
9	Used	Correct	Longitudinal	Correct	Hard	Correct	Bone/antler	Correct	Butt and dorsal surface	Quite certain
10	Used	Correct	Transverse	Correct	Hard	Correct	Bone/antler	Correct	Dorsal surface	Quite certain
11	Used	Correct	Transverse	Correct	Hard	Correct	Indeterminate	Partially correct	Butt and dorsal surface	Quite certain
12	Used	Correct	Transverse	Correct	Soft	Correct	Soft animal material	Correct	Butt	Quite certain
13	Unused	Incorrect	None	Incorrect	None	Incorrect	None	Incorrect	No traces	Quite certain
14	Used	Correct	Transverse	Correct	Medium	Correct	Ligneous vegetal material (soft wood or reeds)	Correct	Butt and dorsal surface	Very certain
15	Used	Correct	Longitudinal	Correct	Hard	Correct	Bone/antler	Correct	Dorsal surface	Quite certain
16	Used	Correct	Indeterminate	Indeterminate	Medium	Partially correct	Indeterminate	Partially correct	Butt and dorsal surface	Uncertain
17	Used	Correct	Longitudinal	Correct	Hard	Correct	Bone/antler	Correct	Butt and dorsal surface	Quite certain
18	Used	Correct	Transverse	Correct	Medium	Partially correct	Abrasive animal material (Hide)	Correct	Butt and dorsal surface	Quite certain
19	Unused	Correct	None	Correct	None	Correct	None	Correct	No traces	Quite certain
20	Used	Correct	Transverse	Correct	Medium	Correct	Ligneous vegetal material (wood?)	Correct	Butt and dorsal surface	Quite certain
21	Used	Correct	Transverse	Correct	Hard	Partially correct	Bone/antler	Partially correct	Dorsal surface	Very certain
22	Used	Correct	Transverse	Correct	Hard	Correct	Indeterminate	Partially correct	Dorsal surface	Uncertain
23	Used	Correct	Transverse	Incorrect	Medium	Partially correct	Ligneous vegetal material (soft wood or reeds)	Partially correct	Butt	Quite certain
24	Unused	Correct	None	Correct	None	Correct	None	Correct	No traces	Very certain
25	Unused	Correct	None	Correct	None	Correct	None	Correct	No traces	Quite certain
26	Used	Correct	Transverse	Correct	Hard	Partially correct	Bone/antler	Partially correct	Butt and dorsal surface	Quite certain
27	Used	Correct	Indeterminate	Indeterminate	Soft	Incorrect	Indeterminate	Indeterminate	Butt and dorsal surface	Uncertain
28	Used	Correct	Transverse	Correct	Soft	Incorrect	Indeterminate	Indeterminate	Butt and dorsal surface	Uncertain
29	Used	Correct	Transverse	Correct	Hard	Partially correct	Bone/antler	Partially correct	Butt and dorsal surface	Quite certain
30	Used	Correct	Transverse	Correct	Medium	Partially correct	Abrasive animal material (Dry hide)	Correct	Butt and dorsal surface	Very certain
31	Unused	Correct	None	Correct	None	Correct	None	Correct	No traces	Quite certain
32	Used	Correct	Indeterminate	Indeterminate	Soft	Incorrect	Soft animal material (meat, fresh hide)	Incorrect	Butt and dorsal surface	Quite certain
33	Unused	Correct	None	Correct	None	Correct	None	Correct	No traces	Uncertain
34	Used	Correct	Longitudinal	Incorrect	Soft	Incorrect	Soft animal material	Incorrect	Butt and dorsal surface	Uncertain
35	Unused	Correct	None	Correct	None	Correct	None	Correct	No traces	Uncertain
36	Unused	Incorrect	None	Incorrect	None	Incorrect	None	Incorrect	No traces	Quite certain
37	Used	Correct	Transverse	Correct	Hard	Incorrect	Indeterminate	Indeterminate	Dorsal surface	Uncertain
38	Used	Correct	Indeterminate	Indeterminate	Indeterminate	Indeterminate	Indeterminate	Indeterminate	Indeterminate	Uncertain
39	Unused	Correct	None	Correct	None	Correct	None	Correct	No traces	Quite certain
40	Unused	Correct	None	Correct	None	Correct	None	Correct	No traces	Quite certain

**Table 5:** The responses and correctness of Blind Tester 2.

Flake number	Used/Unused		Directionality		Contact material-general		Contact material—specific		Location of traces	Level of certainty
	Blind Tester Response	Correctness of response	Blind Tester Response	Correctness of response	Blind Tester Response	Correctness of response	Blind Tester Response	Correctness of response		
1	Unused	Incorrect	None	Incorrect	None	Incorrect	None	Incorrect	No traces	Quite certain
2	Used	Correct	Indeterminate	Indeterminate	Hard/medium	Incorrect	Plant medium-hard undetermined	Incorrect	Butt	Quite certain
3	Used	Correct	Transverse	Incorrect	Hard/medium	Incorrect	Plant medium-hard undetermined	Partially correct	Butt and dorsal surface	Quite certain
4	Used	Correct	Transverse	Correct	Hard/medium	Correct	Abrasive unsure	Partially correct	Butt	Quite certain
5	Used	Correct	Transverse	Incorrect	Medium	Incorrect	Hide/ indeterminate	Partially correct	Dorsal surface	Very certain
6	Unused	Incorrect	None	Incorrect	None	Incorrect	None	Incorrect	No traces	Quite certain
7 -Flake missing	-	-	-	-	-	-	-	-	-	-
8	Used	Correct	Transverse	Correct	Hard/medium	Correct	Indeterminate	Indeterminate	Dorsal surface	Uncertain
9	Used	Correct	Longitudinal	Correct	Medium	Incorrect	Hide +additive	Incorrect	Butt and dorsal surface	Very certain
10	Used	Correct	Transverse	Correct	Hard	Partially correct	Bone	Incorrect	Butt	Quite certain
11	Unused	Incorrect	None	Incorrect	None	Incorrect	None	Incorrect	No traces	Quite certain
12	Unused	Incorrect	None	Incorrect	None	Incorrect	None	Incorrect	No traces	Quite certain
13	Used	Correct	Longitudinal	Correct	Hard	Correct	Bone	Correct	Dorsal surface	Quite certain
14	Used	Correct	Transverse	Correct	Hard	Partially correct	Bone	Partially correct	Butt and dorsal surface	Very certain
15	Used	Correct	Longitudinal	Correct	Hard	Correct	Bone	Correct	Dorsal surface	Quite certain
16	Unused	Incorrect	None	Incorrect	None	Incorrect	None	Incorrect	No traces	Uncertain
17	Used	Correct	Longitudinal	Correct	Hard	Correct	Indeterminate	Indeterminate	No traces	Uncertain
18	Unused	Incorrect	None	Incorrect	None	Incorrect	None	Incorrect	No traces	Uncertain
19	Unused	Correct	None	Correct	None	Correct	None	Correct	No traces	Quite certain
20	Used	Correct	Transverse	Correct	Hard/medium	Correct	Plant wood	Correct	Butt and dorsal surface	Quite certain
21	Used	Correct	Transverse	Correct	Hard	Correct	Wood	Correct	Butt and dorsal surface	Very certain
22	Used	Correct	Transverse	Correct	Hard/medium	Correct	Bone/antler?	Correct	Butt	Quite certain
23	Used	Correct	Indeterminate	Indeterminate	Medium?	Partially correct	Indeterminate	Indeterminate	Dorsal surface	Uncertain
24	Unused	Correct	None	Correct	None	Correct	None	Correct	No traces	Quite certain
25	Unused	Correct	None	Correct	None	Correct	None	Correct	No traces	Quite certain
26	Used	Correct	Transverse	Correct	Hard	Correct	Bone	Partially correct	Dorsal surface	Very certain
27	Unused	Incorrect	None	Incorrect	None	Incorrect	None	Incorrect	No traces	Quite certain
28	Unused	Incorrect	None	Incorrect	None	Incorrect	None	Incorrect	No traces	Quite certain
29	Used	Correct	Transverse	Correct	Hard/medium	Correct	Plant medium-hard indeterminate	Correct	Butt and dorsal surface	Very certain
30	Used	Correct	Transverse	Correct	Medium	Incorrect	Abrasive indeterminate (hide)	Correct	Butt	Very certain
31	Unused	Correct	None	Correct	None	Correct	None	Correct	No traces	Uncertain
32	Unused	Incorrect	None	Incorrect	None	Incorrect	None	Incorrect	No traces	Quite certain
33	Used	Incorrect	Indeterminate	Indeterminate	Medium	Incorrect	Indeterminate	Indeterminate	Dorsal surface	Uncertain
34	Unused	Incorrect	None	Incorrect	None	Incorrect	None	Incorrect	No traces	Uncertain
35	Unused	Correct	None	Correct	None	Correct	None	Correct	No traces	Uncertain
36	Unused	Incorrect	None	Incorrect	None	Incorrect	None	Incorrect	Dorsal surface	Uncertain
37	Used	Correct	Indeterminate	Indeterminate	Hard	Incorrect	Indeterminate	Indeterminate	Butt	Quite certain
38	Unused	Incorrect	None	Incorrect	None	Incorrect	None	Incorrect	No traces	Quite certain
39	Unused	Correct	None	Correct	None	Correct	None	Correct	Dorsal surface	Uncertain
40	Unused	Correct	None	Correct	None	Correct	None	Correct	Butt	Uncertain

**Table 6:** The responses and correctness of Blind Tester 3.

Flake number	Used/Unused		Directionality		Contact material-general		Contact material—specific		Location of traces	Level of certainty
	Blind Tester Response	Correctness of response	Blind Tester Response	Correctness of response	Blind Tester Response	Correctness of response	Blind Tester Response	Correctness of response		
1	Used	Correct	Longitudinal	Incorrect	Medium	Incorrect	Hide?	Correct	Butt and dorsal surface	Uncertain
2	Used	Correct	Transverse	Correct	Soft	Correct	Hide	Correct	Dorsal surface	Uncertain
3	Used	Correct	Longitudinal	Correct	Soft	Correct	Plant?	Correct	Butt and dorsal surface	Quite certain
4	Used	Correct	Transverse	Correct	Hard	Correct	Bone	Correct	Butt	Quite certain
5	Used	Correct	Longitudinal	Correct	Hard	Correct	Bone	Correct	Butt and dorsal surface	Very certain
6	Used	Correct	Transverse	Correct	Soft	Incorrect	Plant	Partially correct	Dorsal surface	Quite certain
7	Used	Correct	Indeterminate	Indeterminate	Hard	Incorrect	Bone?	Incorrect	Dorsal surface	Quite certain
8	Used	Correct	Longitudinal	Incorrect	Medium	Correct	Wood/plant	Partially correct	Butt and dorsal surface	Uncertain
9	Used	Correct	Transverse	Incorrect	Medium	Incorrect	Wood	Partially correct	Butt and dorsal surface	Uncertain
10	Used	Correct	Longitudinal	Incorrect	Hard	Incorrect	Bone	Incorrect	Butt and dorsal surface	Very certain
11	Used	Correct	Transverse	Correct	Hard	Correct	Bone?	Correct	Butt	Uncertain
12	Used	Correct	Transverse	Correct	Medium	Incorrect	Wood	Incorrect	Butt and dorsal surface	Uncertain
13	Used	Correct	Longitudinal	Correct	Hard	Correct	Bone	Correct	Butt and dorsal surface	Quite certain
14	Used	Correct	Transverse	Correct	Medium	Correct	Wood	Correct	Butt and dorsal surface	Very certain
15	Unused	Incorrect	None	Incorrect	None	Incorrect	None	Incorrect	No traces	
16	Used	Correct	Transverse	Incorrect	Medium	Incorrect	Wood	Incorrect	Dorsal surface	Quite certain
17	Used	Correct	Longitudinal	Correct	Hard	Correct	Bone	Correct	Dorsal surface	Very certain
18	Used	Correct	Transverse	Correct	Soft	Correct	Indeterminate	Indeterminate	Dorsal surface	Uncertain
19	Unused	Correct	None	Correct	None	Correct	None	Correct	No traces	Certain
20	Used	Correct	Transverse	Correct	Soft	Incorrect	Plant?	Partially correct	Butt and dorsal surface	Uncertain
21	Used	Correct	Transverse	Correct	Medium	Correct	Wood/plant	Correct	Butt and dorsal surface	Quite certain
22	Used	Correct	Longitudinal	Incorrect	Soft	Incorrect	Butchering?	Partially correct	Dorsal surface	Uncertain
23	Used	Correct	Transverse	Incorrect	Medium	Incorrect	Wood	Incorrect	Dorsal surface	Quite certain
24	Unused	Correct	None	Correct	None	Correct	None	Correct	No traces	Certain
25	Unused	Correct	None	Correct	None	Correct	None	Correct	No traces	Certain
26	Used	Correct	Transverse	Correct	Medium	Correct	Wood	Correct	Butt and dorsal surface	Very certain
27	Used	Correct	Transverse	Correct	Soft	Incorrect	Indeterminate	Indeterminate	Butt and dorsal surface	Uncertain
28	Used	Correct	Transverse	Correct	Medium	Correct	Indeterminate	Indeterminate	Butt and dorsal surface	Uncertain
29	Used	Correct	Transverse	Correct	Hard	Partially correct	Bone	Partially correct	Dorsal surface	Quite certain
30	Used	Correct	Transverse	Correct	Soft	Correct	Hide	Correct	Butt and dorsal surface	Very certain
31	Unused	Correct	None	Correct	None	Correct	None	Correct	No traces	Certain
32	Used	Correct	Transverse	Correct	Medium	Correct	Indeterminate	Indeterminate	Butt	Uncertain
33	Unused	Correct	None	Correct	None	Correct	None	Correct	No traces	Certain
34	Used	Correct	Transverse	Correct	Medium	Correct	Wood?	Correct	Butt and dorsal surface	Uncertain
35	Used	Incorrect	Transverse	Incorrect	Hard?	Incorrect	Indeterminate	Incorrect	Butt and dorsal surface	Uncertain
36	Unused	Incorrect	None	Incorrect	None	Incorrect	None	Incorrect	No traces	Certain
37	Used	Correct	Transverse	Correct	Medium	Incorrect	Wood	Incorrect	Butt	Quite certain
38	Used	Correct	Transverse	Incorrect	Medium	Incorrect	Indeterminate	Indeterminate	Butt and dorsal surface	Uncertain
39	Unused	Correct	None	Correct	None	Correct	None	Correct	No traces	Certain
40	Unused	Correct	None	Correct	None	Correct	None	Correct	No traces	Certain

For the variables “Contact material–General” and “Contact material–Specific” the supporting notes of blind testers were taken into consideration with answers being scored as “Partially correct” in cases where the answer that was provided was close to the correct answer, e.g. bone traces being mistaken for antler traces, or where the answer was supported by reasoning that indicated that the blind tester was clearly thinking in the right direction. For example, in their notes for Flake 5 Blind Tester 2 stated that they identified traces that they believed were related to bone-working, which was the correct use of the tool, but erroneously believed that the primary use of the tool related to hide working. As a result they gave a response of “Hide/ indeterminate” to the variable Contact material—Specific. This answer was scored as “Partially correct” on the basis that they had identified the correct traces in the notes, but also incorrectly identified other traces which were recorded as the main response. The notes of the blind testers can be found in S1 Table.

**Fig 3:**
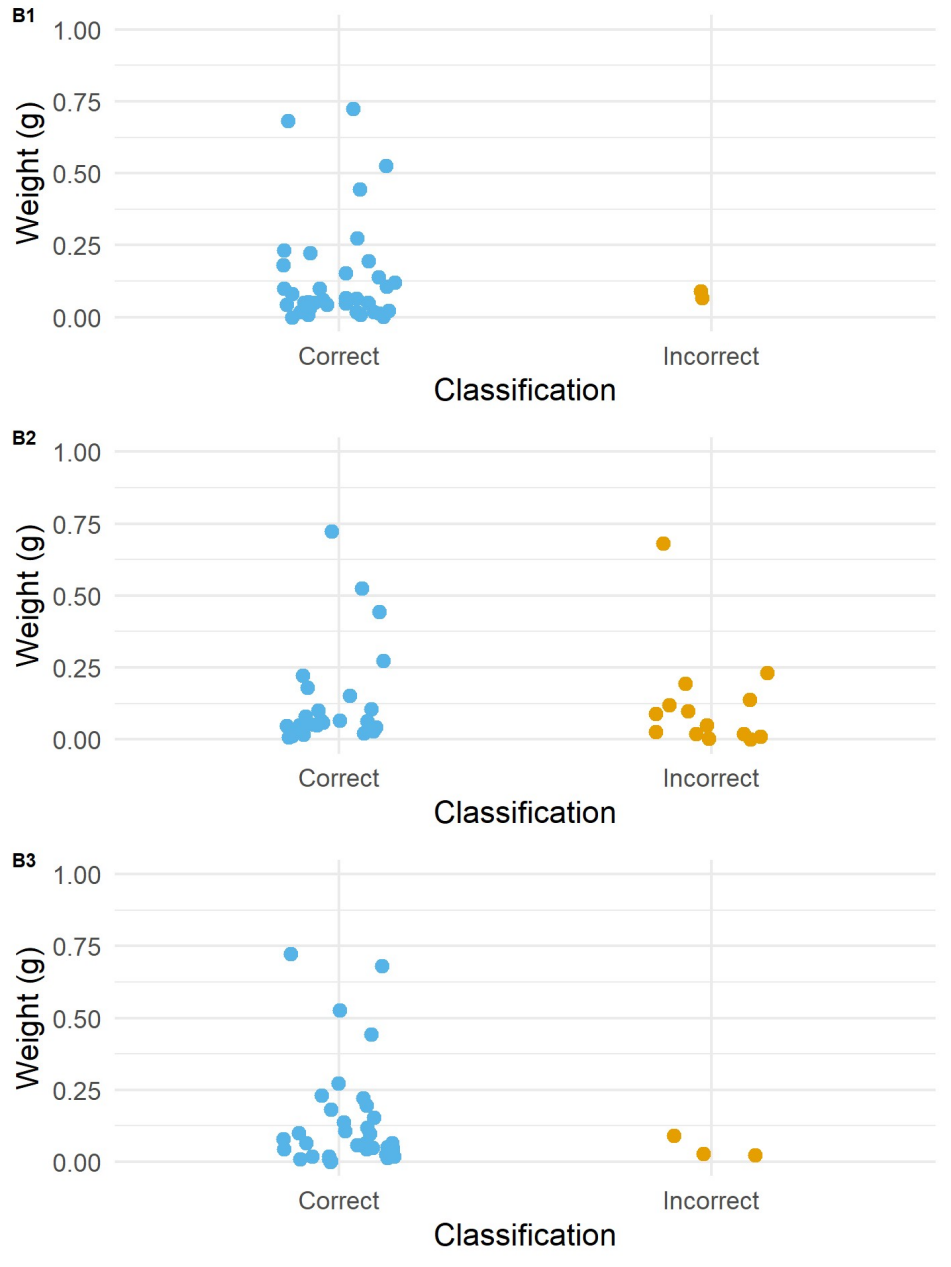
Jitter plots for each blind tester showing the dispersion of correct and incorrect responses to the used/unused attribute according to flake weight (g). Blind Tester number indicated in top left hand corner of each plot.

**Fig 4:**
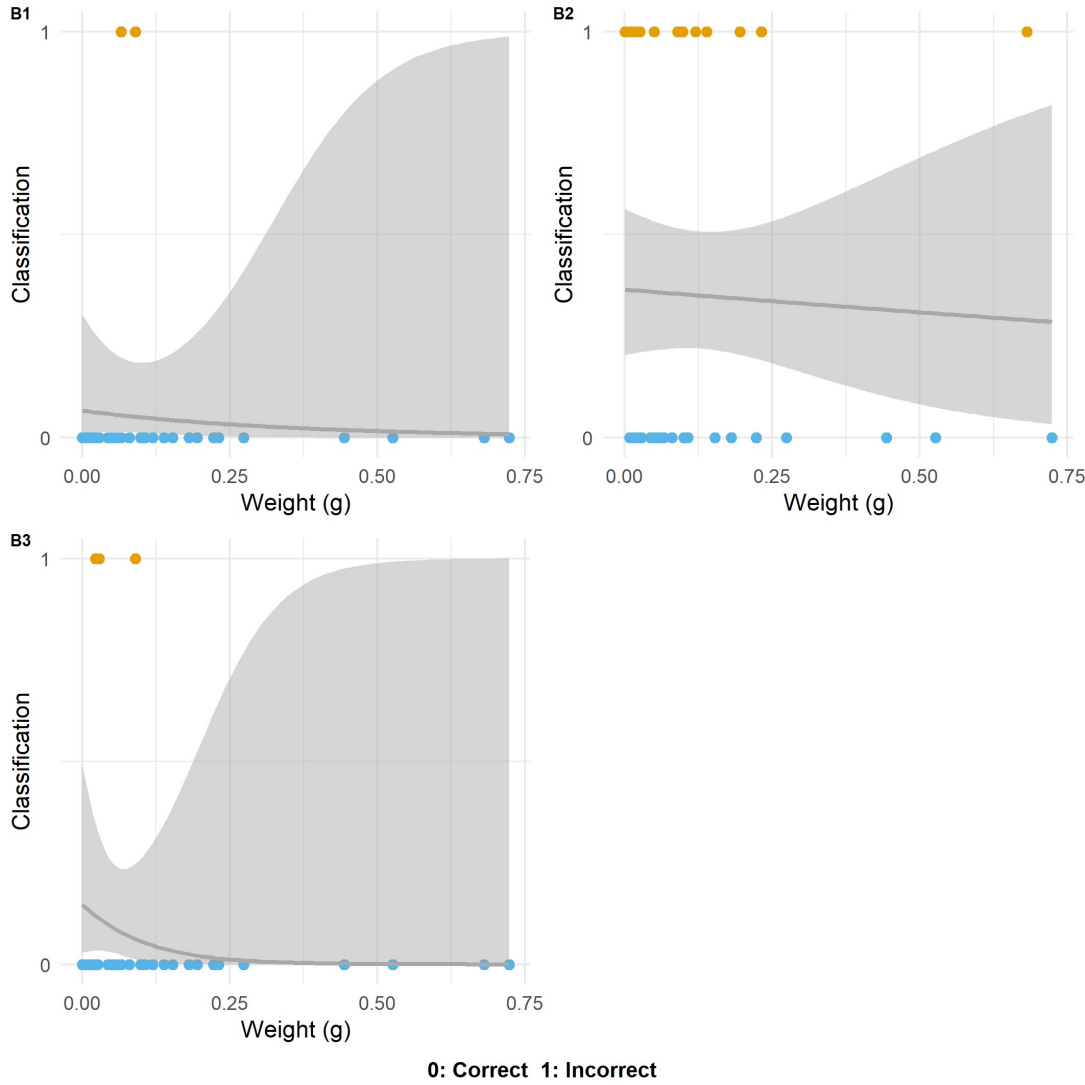
Logistic regression plots for each blind tester showing the relationship between flake weight and correct and incorrect responses. Blind Tester number indicated in top left hand corner of each plot.

In order to examine the relationship between the weight of flakes and the proportion of correct or incorrect answers for the used/unused attribute, responses were scored as 0 if correct and 1 if incorrect with data being separated out for each Blind Tester. Measures of dispersion among the weighting of responses were first examined through a jitter plot to avoid overplotting in discrete positions (Fig 3). Generalized Linear Models (GLMs) of binomial class were then constructed to create Logistic Regression plots for each individual (Fig 4). Parameter significance values and pseudo p-values (likelihood ratio p-values) were then calculated for each GLM. In each circumstance, a null hypothesis (H_0_) of same populations (weight) between incorrect and correct responses for each participant was assumed, and an alpha level of 0.05 was adopted given the number of artefacts examined (n = 40). Log-likelihood values, chi-square values (include deviance and null-deviance values) and pseudo r-square values, through a Hosmer and Lemeshow statistical test for goodness of fit [35], are supplied as additional data in the article code which can be found on GitHub and the Open Science Framework (see below for repository information).

All graphics and analyses were developed in either SPSS v.24, or the R Environment [36], using the *tidyverse* [37], *rms* [38] and *ggpubr* [39] packages. In promoting data transparency and replicability in archaeological analyses [40] the blind test data is contained in Tables 4–6, and available as an Excel spreadsheet in the S1 Table, and additional data and code used to create Figs 3, 4 and 14 can be found on GitHub (https://github.com/CSHoggard/-use_wear_test) and on the Open Science Framework (https://osf.io/4k5j6/).

## Results

Before discussing the results of the blind test, it is necessary to note some observations made during the selection procedure for the blind test sample. Most surprising was how many of the resharpening flakes were found to lack identifiable wear traces during their assessment prior to the sample selection. The principal reason for this was the crushing of the butts of the resharpening flakes during retouching, which obliterated the external platform edge of the flakes where use-traces are most likely to occur. Even if just the resharpening flakes with surviving butts are considered; from the retouching of 16 tools 139 retouch flakes with surviving butts were produced, of which 32 were considered during initial assessment to have relatively good surviving wear-traces. In this case, “good” does not mean they were easily interpretable, just that traces were present to enough of an extent that a potential for interpretation was provided. These results clearly highlight the fact that whilst the *presence* of wear traces on resharpening flakes may indicate that the flake comes from a *used* tool, the *absence* of traces does not unequivocally indicate that the flake is struck from an *unused* tool. These flakes may equally be struck from an unused part of an edge of an otherwise used tool, or have had existing wear traces obliterated by the hammer blow during resharpening. This has important ramifications for the interpretation of archaeological assemblages of retouch flakes, with a clear potential for the presence of resharpening flakes to be underrepresented during analysis.

### Used/unused

**Fig 5:**
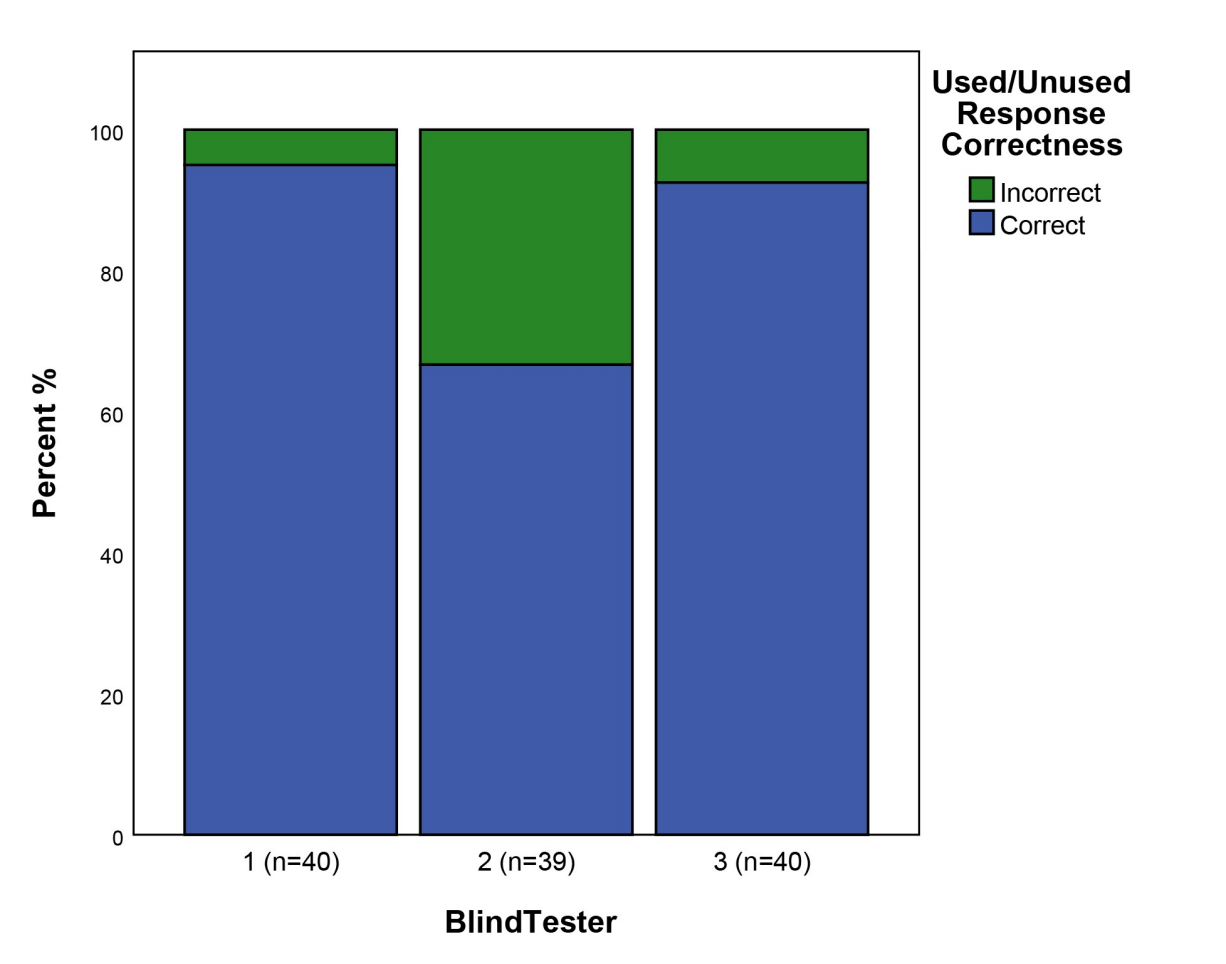
The correctness of responses to the used/unused attribute.

During the scoring of the blind test flakes from used tools that were struck from an unused part of the edge of the tool were counted as “unused”. In total therefore this means that 32 flakes were from used tools/edges, and 8 were from unused tools/edges. Overall there was a high level of accuracy in the assessment of used/unused, with blind testers providing the correct answer on average 85% of the time across the 3 blind testers (highest 95% correct n = 40, lowest = 67% correct n = 40) (Fig 5). There was some variability between testers, with two testers scoring highly in terms of accuracy, and one scoring lower. There was slight correlation with analyst experience with Blind Tester 2 (66.7% correct n = 39) having the least years of use-wear analysis experience of the three testers.

### Directionality

**Fig 6:**
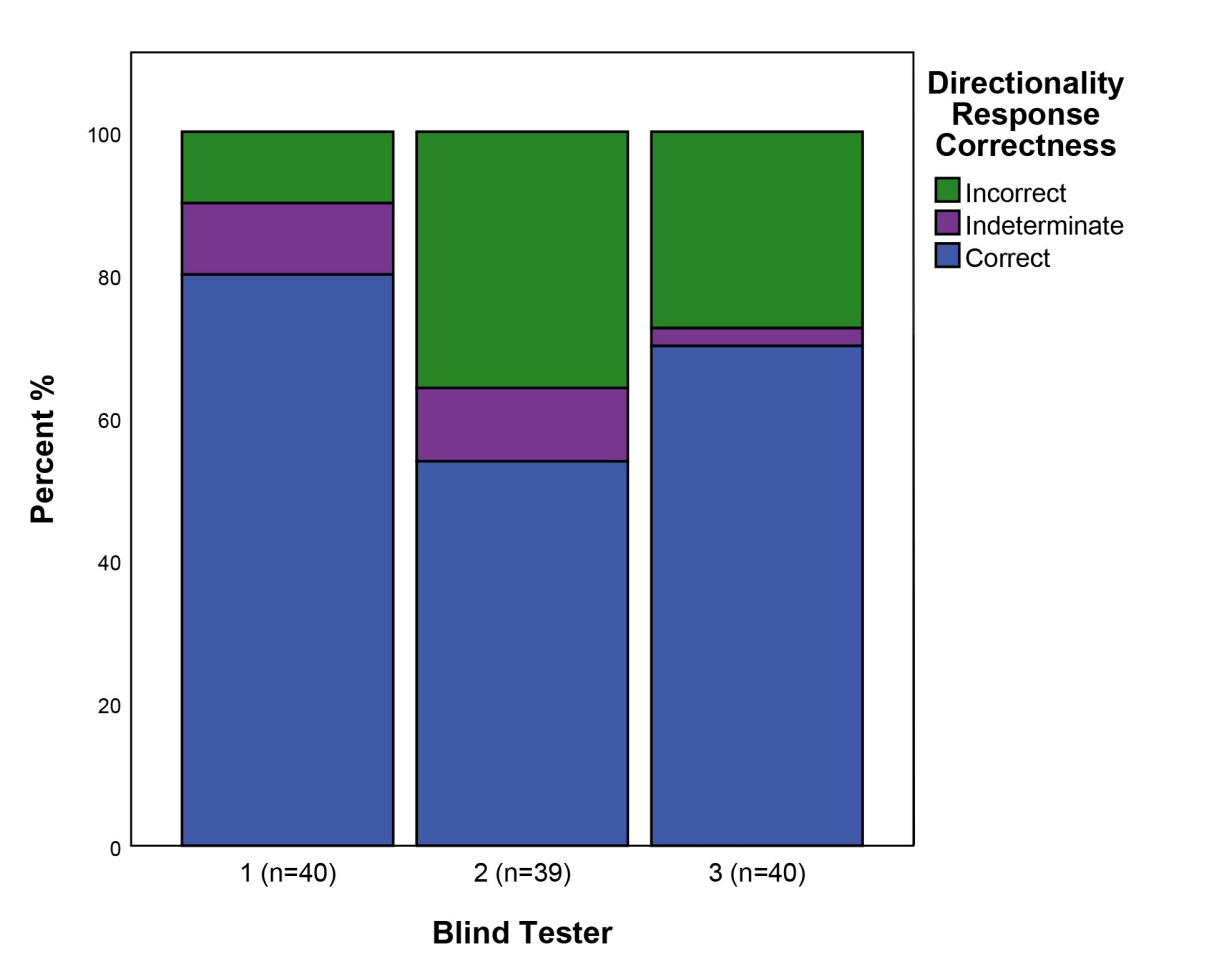
The correctness of responses to the directionality attribute. See Table 3 for a description of attribute states.

As with the Used/Unused attribute, in scoring directionality flakes from the unused edges of used tools were treated as being from unused tools having no directionality. Taking this into account, 22.5% of the blind test flakes came from tools used longitudinally, 57.5% from tools used transversely, and 20% from unused tools/edges. As mentioned above, the pre-test assessment checked for signs of use-wear, but did not check for traces indicating their directionality of use. Therefore 100% accuracy for this attribute was not necessarily achievable. Perhaps reflecting this, the identification of the directionality of use shows a slightly lower degree of accuracy than that of used/unused, with directionality being correctly identified on average 68% of cases, being recorded as indeterminate in 8% of cases, and being incorrectly identified in 24% of cases (Fig 6). There is a clear correlation between the accuracy for this attribute and that for used/unused with the same Blind Testers having the highest and lowest levels of accuracy in both cases (Blind Tester 1 80% correct n = 40, Blind Tester 2 54% correct n = 39).

**Fig 7:**
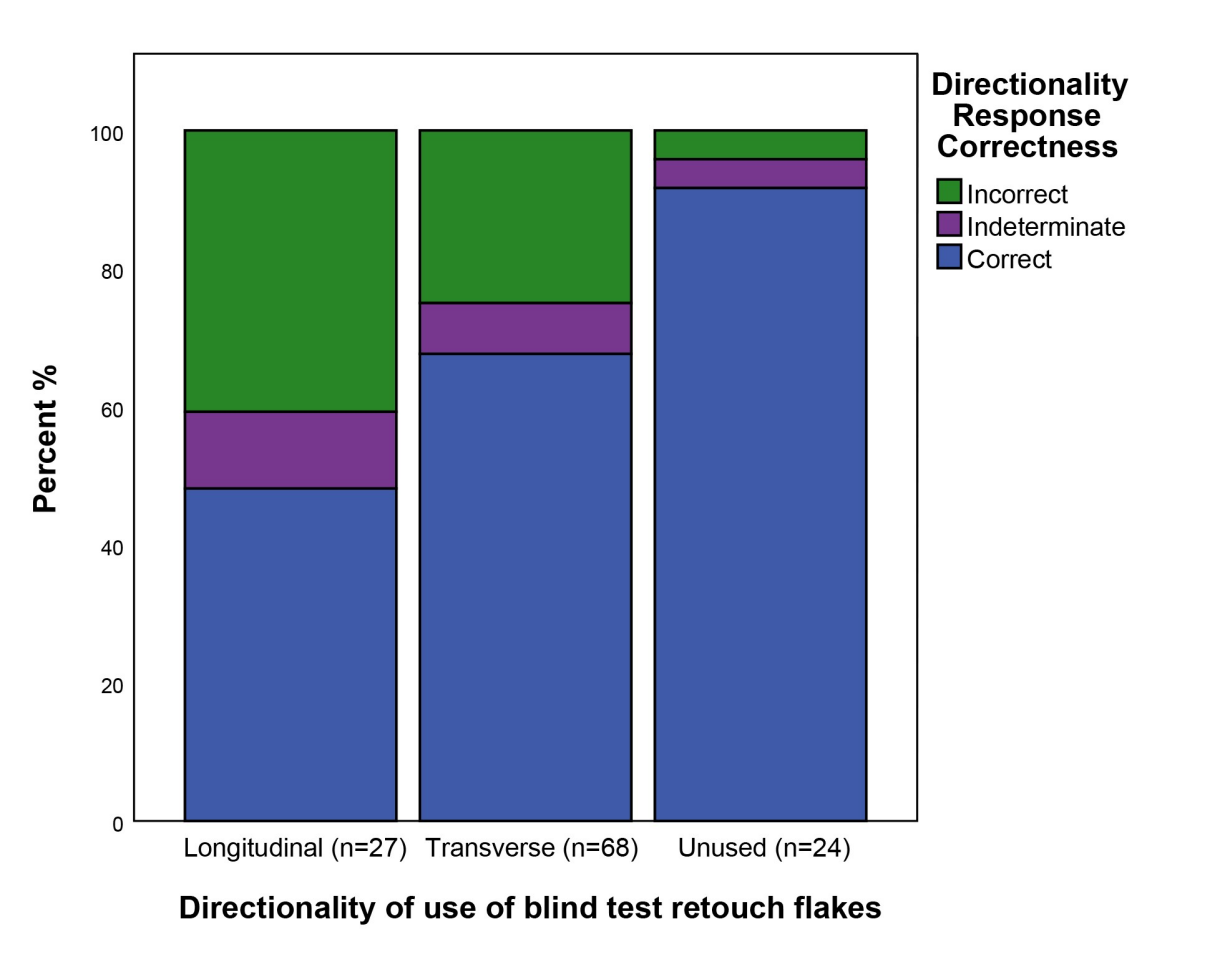
The correctness of responses split by the directionality of use of retouch flakes. See Table 3 for a description of attribute states.

If the correctness of responses are broken down according to the directionality of use of individual flakes, there is variability in accuracy between flakes with differing directionalities of use (Fig 7). Accuracy was far greater in the case of unused tools which have no directionality, whilst amongst used flakes transverse directionalities (68% correct n = 68) were correctly identified more frequently than longitudinal directionalities (48% correct n = 27).

### Contact material—general

This variable sought to assess accuracy in identifying the general type of contact material that tools were used on. The variable states are commonly used terms within use-wear analysis defining whether the hardness of the contact material was soft (e.g. fresh hide), medium (e.g. woody plants), or hard (e.g. bone). There is a tacit, rather than explicit, agreement between use-wear analysts about which materials are deemed to be soft, medium, or hard. In practice the definition of these categories relies on observations of the character of wear traces and particularly the distribution of traces in relation to the used edge and surface micro-topography. For example, wear traces from contact with softer materials generally have diffuse boundaries and are more likely to penetrate into the low parts of the micro-topography of the surface of a tool, also penetrating further into the interior of the tool away from the use-edge [34 p27-41, 10 Ch. 3].

**Fig 8:**
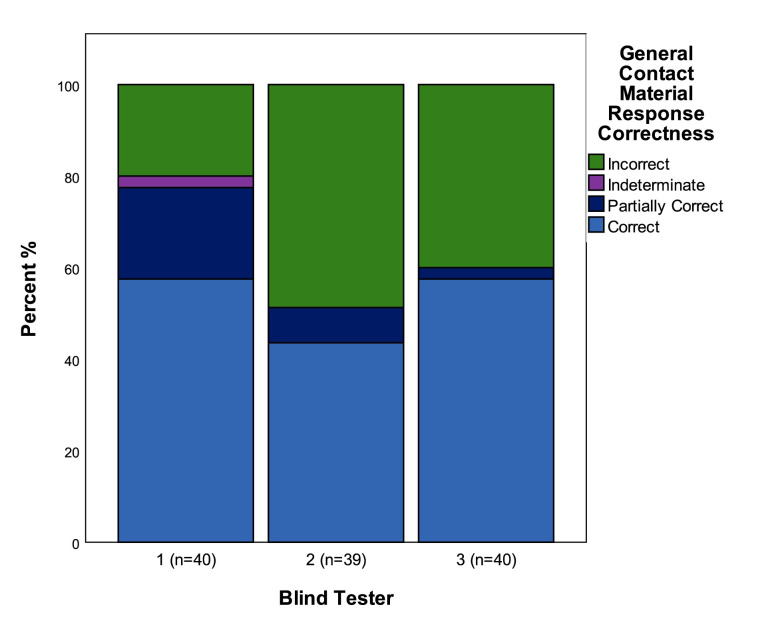
The correctness of identifications of general categories of contact material.

Between the three Blind Testers general categories of contact materials were correctly identified in 53% of cases (n = 119), and partially correctly identified in a further 10% of cases. Blind Testers 1 and 3 (n = 40) had the highest levels of accuracy with 58% correct determinations, Blind Tester 1 had a further 20% of partially correct determinations, with Blind Tester 3 having 2.5% partially correct determinations (Fig 8).

### Contact material—specific

**Fig 9:**
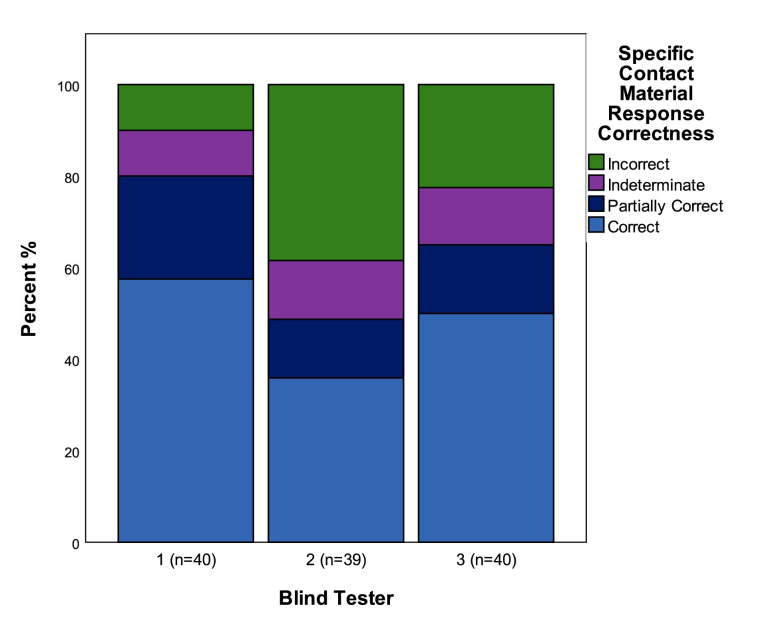
The correctness of identifications of specific categories of contact material.

This variable asked the tester to define the specific contact material that a tool was used on. Given the range of potential answers no predetermined variable states could be defined and therefore some latitude is required in scoring the correctness of the answers. When averaging the results of all three testers the correct contact material, or lack of in the case of unused tools, was identified in 48% of cases with 17% of determinations being partially correct. The highest level of accuracy was recorded by Blind Tester 1 (n = 40) who was correct in 58% of cases, partially correct in 23% of cases, with a further 10% being classed as indeterminate. Blind Tester 2 (n = 39) had the lowest level of accuracy with 36% of determinations being correct, with a further 13% being partially correct, and 13% being indeterminate (Fig 9). Given that the identification of the specific contact material that a tool was used upon was analytically the most difficult task set for the blind test, the lower average level of accuracy for this variable was expected. Bearing this in mind it is remarkable that Blind Tester 1 exhibited the same level of accuracy as they had for determining the general category of contact material.

In analytical terms, it is also worth considering that partially correct or indeterminate identifications are not necessarily problematic in that they will lead to a lack of interpretable results, as opposed to results that are actually wrong. Therefore, it is perhaps equally useful to examine how often contact materials were incorrectly identified. In this respect the average proportion of incorrect determinations between all testers was 24% with the lowest rate being 10% for Blind Tester 1, and the highest being 39% for Blind Tester 2. This indicates that in the majority of cases analysis will lead to conclusions that are correct, or limited by uncertainty, rather than wrong.

### The effect of contact material on accuracy

**Fig 10:**
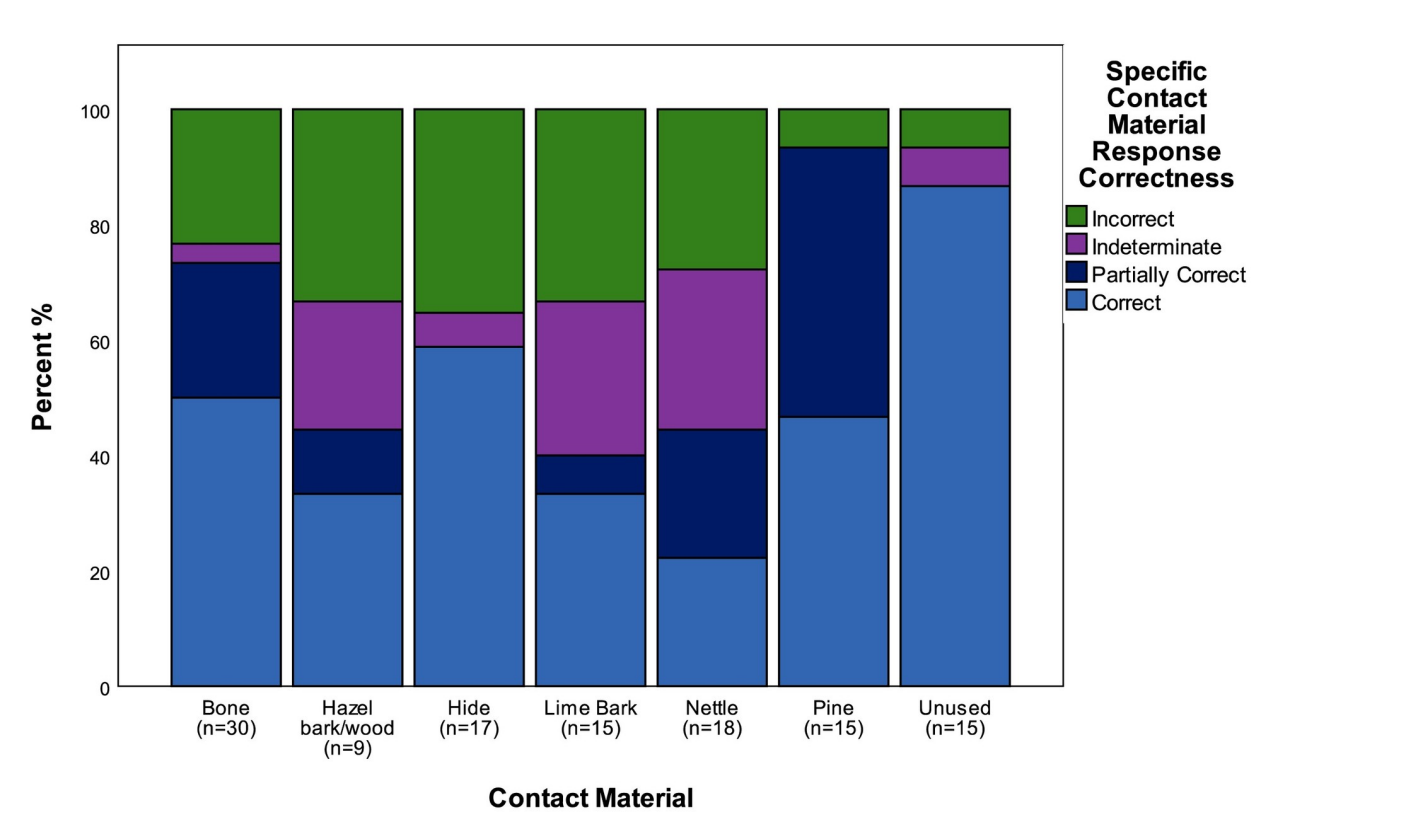
The correctness of identifications for all blind testers split by contact materials.

**Fig 11:**
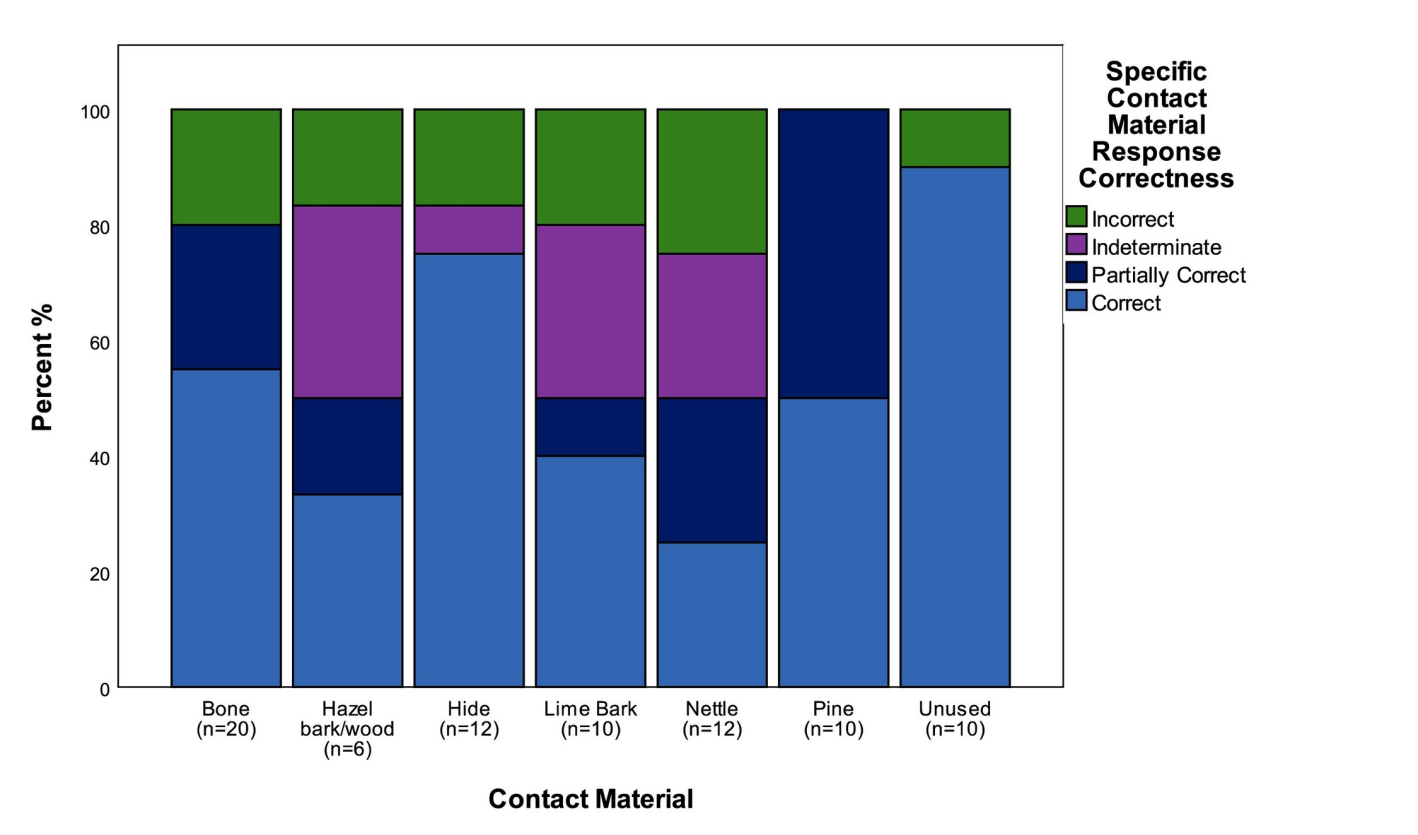
The correctness of identifications for Blind Testers 1 and 3 split by contact materials.

An important question in understanding the levels of accuracy with which traces were identified on retouch flakes is whether it was affected by the directionality with which the parent tools were used, the size of the retouch flakes, the type of hammer used to remove them, or by the type of contact material the tools were used on. The latter can be assessed by examining the proportion of correct and incorrect determinations for each of the six different contact materials and the flakes from unused edges (Fig 10). Regardless of the overall levels of accuracy of individual testers, the data shows a consistent pattern in that retouch flakes from unused edges, and those from tools used on pine, bone and hide were correctly identified more frequently. Those used on nettle, lime bark, and hazel branches, caused the most problems in identification. There are multiple causal factors behind incorrect identifications, in order to eliminate some of this variability it is worth examining responses from the two testers with the highest overall levels of accuracy (Fig 11). This shows that the contact material that these two testers had difficulty in identifying where exactly the same as for all testers as a whole, confirming the previous pattern, and suggesting that there is something about the traces themselves that made identification more difficult.

The experiments on hide, bone and pine all produced quite highly characteristic traces that all experienced use-wear analysts are familiar with, and particularly in the case of bone, traces that are characteristic even when only small patches of polish are present (Fig 2). In contrast the experiments on lime bark and nettle stems did not produce such strong wear traces, necessitating in both cases that the duration of the experiments be continued beyond the standard 90 minutes in order to produce traces that were readily identifiable on the intact tool edges, let alone on the retouch flakes. In this respect it can be expected that if the original experiments were all conducted for longer durations the level of accuracy would have risen, and equally so if the blind test had been limited to tools used on bone, pine and hide. Beyond that, it is worth noting that the traces that the Testers had problems with were those which they had little experience of analysing prior to the test, particularly in the case of nettles and lime bark. This therefore has some ramifications for the identification of contact materials in archaeological contexts. It can be expected that analysing unfamiliar traces requires piecing together an interpretation based on a range of wear trace characteristics, such as the extent of edge rounding, character of use-polish, and location of the polish in relation to the microtopography and edge morphology. This is a necessarily more difficult task where only a small portion of the working edge of the tool is available for analysis, as is the case when studying retouch flakes. Alternatively, when dealing with the small surfaces available when analysing retouch flakes, the identification of traces that are familiar and well-known to the analyst can be expected to be an easier proposition.

### The effect of directionality on accuracy

Between the three blind testers the directionality of use of the parent tool was correctly identified on retouch flakes 68% of the time, with 8% of cases being indeterminate, and 24% being incorrectly identified. The main question is what affected the blind tester’s ability to recognise directionality on the retouch flakes. Within the two main modes of directionality, transverse motions were correctly identified 68% of the time and longitudinal motions were correctly identified 48% of the time. In other words, it was easier to determine wear traces related to scraping motions than to cutting motions. These results are in keeping with studies of polish development with Vaughan [34 p29] noting that due to edge morphology and the kinetics of different types of motions, activities involving sawing motions tend disperse contact over a large area of the use-edge, whereas transverse motions subject a smaller part of the edge of a tool to more intense and sustained contact. Thus wear traces indicating the direction of use of a tool are likely to be generated more rapidly on scraping tools than on cutting tools. On the retouched edges of tools used in a longitudinal motion traces tend to develop initially and more strongly on areas of higher topography, such as the arrises between retouch flake scars. Therefore, in comparison with transversally used tools such as scrapers where the flat ventral side of the tool is in contact with the material being worked, traces from longitudinal working are spread more widely along the use-edge but occur in more localised spots.

The differences in the development of wear traces between tools used in a longitudinal or transverse motion would therefore appear to explain the data for directionality within the blind test. A cross tabulation of the correctness of response for directionality with contact material, however, suggests that the situation is more complex (Table 7). The data indicate that the proportion of correct responses for directionality was highest among retouch flakes from unused tools, with the proportion of correct responses for retouch flakes from tools used on wood, bone and hide being comparable with one another, varying from 65% to 74%. The type of contact material that caused the most difficulties was plant, with directionality being correctly identified in only 39% of cases.

**Table 7:** Cross tabulation of the correctness of response for directionality with general categories of contact material.

Correctness of response for Directionality		Contact Material					Total
		Bone	Hide	Plant	Unused	Wood	
**Correct**	Count	21	11	7	13	29	81
	% within Contact Material	70.0%	64.7%	38.9%	86.7%	74.4%	68.1%
**Incorrect**	Count	9	4	7	1	8	29
	% within Contact Material	30.0%	23.5%	38.9%	6.7%	20.5%	24.4%
**Indeterminate**	Count	0	2	4	1	2	9
	% within Contact Material	0.0%	11.8%	22.2%	6.7%	5.1%	7.6%
**Total**	Count	30	17	18	15	39	119
	% within Contact Material	100.0%	100.0%	100.0%	100.0%	100.0%	100.0%

The lower rates of correct identifications of directionality amongst retouch flakes from tools used to work plants raises the possibility that it is the contact material, rather than the direction of use of the tool, that had the biggest effect on accuracy for this variable. Understanding whether this was the case is complicated by the fact that only the bone and plant working experiments involved using a tool in a longitudinal motion. A cross tabulation of the directionality of tool use with the correctness of blind tester responses for directionality shows crucial differences between the two materials. The blind testers found it significantly easier to identify directionality on retouch flakes from tools used to work bone longitudinally than they did on plant working tools used with the same motion (Table 8). This indicates that it is not just the direction of tool use, but also the type of contact material, that effects the recognition of traces of directionality. The likely reason for this relates to differences in the way in which wear traces from hard (bone) and soft (plant) contact materials develop on the edges of tools. Due to its hardness, wear traces from contact with bone tend to be restricted to higher areas of topography. As a result the traces that are generated are often localised but also intensely developed (micrograph E in Fig 2). Polish development from contact with softer materials, such as plants, occurs more slowly with traces tending to be more diffuse. Therefore traces of longitudinal directionality on retouched edges used to work bone may be localised but, if spotted, easily identifiable, whereas comparable traces on plant working tools may be spread more diffusely and harder to identify. This has obvious ramifications when only a small portion of the use-edge is available for analysis, as is the case for retouch flakes.

**Table 8:** Cross tabulation comparing the directionality of tool use with the correctness of blind tester responses to identifying directionality on retouch flakes from tools used to work bone and plants.

True directionality of tool use			Correctness of response for Directionality			Total
			Correct	Incorrect	Indeterminate	
**Bone**	**Longitudinal**	Count	11	4	-	15
		% within True Directionality	73.3%	26.7%	-	100.0%
	**Transverse**	Count	7	5	-	12
		% within True Directionality	58.3%	41.7%	-	100.0%
**Plant**	**Longitudinal**	Count	2	7	3	12
		% within True Directionality	16.7%	58.3%	25.0%	100.0%
	**Transverse**	Count	2	0	1	3
		% within True Directionality	66.7%	0.0%	33.3%	100.0%
**Total**		Count	22	16	4	42
		% within True Directionality	52.4%	38.1%	9.5%	100.0%

### The effect of hammer type on accuracy

**Fig 12:**
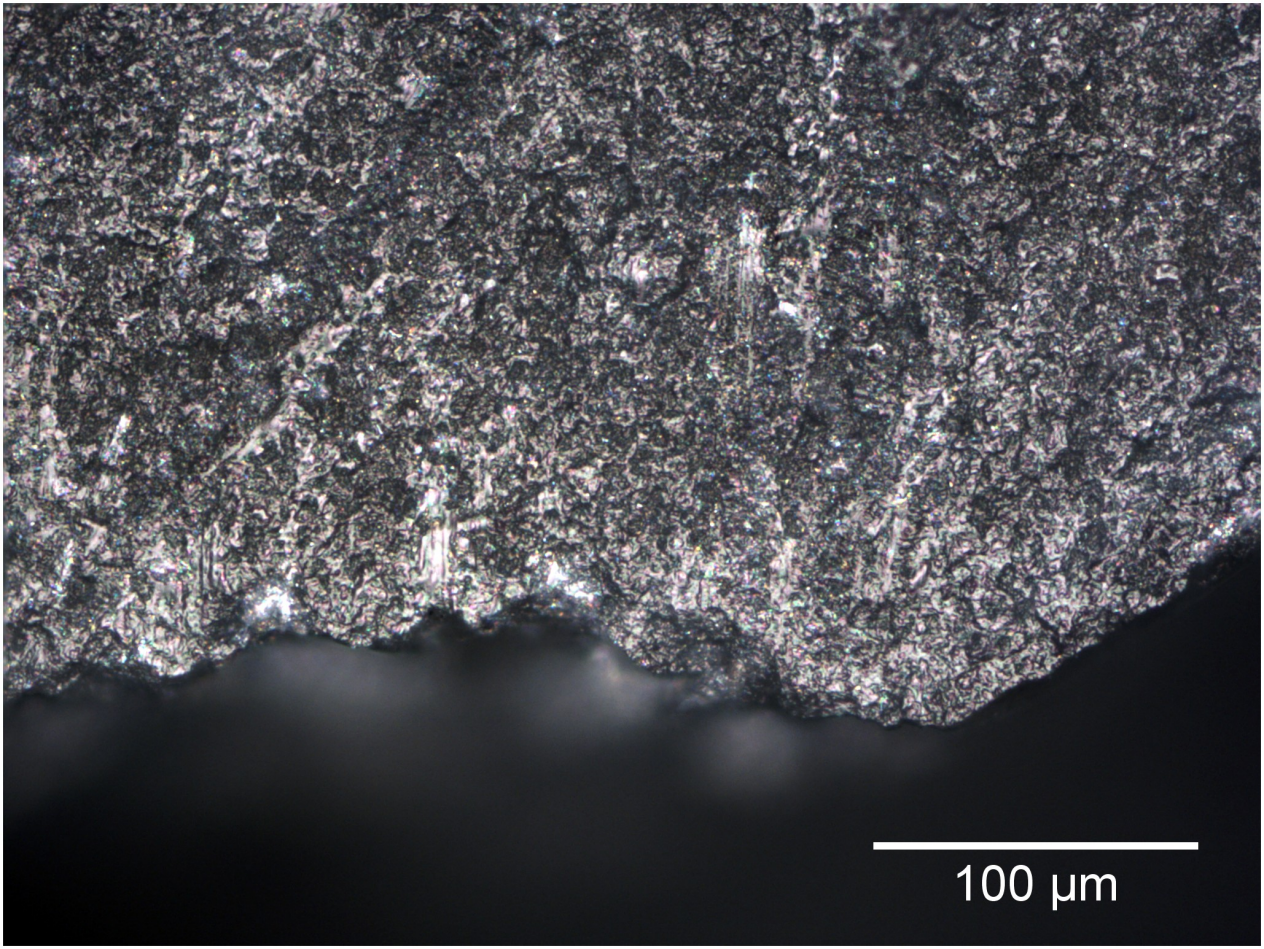
The external platform edge of the butt of Flake 32, a retouch flake removed using a stone hammer from a tool used to scrape lime bark. The diffuse polish near the edge flake is related to the working of the bark, the isolated streaks of flat bright polish associated with linear stria are marks left by the hammer (200x).

Striations and linear streaks of polish from hammers were present on the butts of some of the retouch flakes (Fig 12). A consideration of their effects on the identification of wear traces is therefore warranted. A total of 29 of the blind test retouch flakes were struck with stone hammers, and 11 were struck with antler hammers. Across all blind testers in total there were 87 determinations made on retouch flakes struck with stone hammers, and 32 struck with antler hammers. Cross tabulation of the proportion of correct determinations with hammer type for used-unused, directionality, and general contact material indicates that hammer type made no difference in accuracy for the used-unused category, but correct determinations were 12% less frequent on flakes struck with antler hammers compared to stone hammers for both directionality and general contact material. Whilst this may suggest that antler hammers are more likely to produce traces that are confused with tool use, a closer look at the data reveals that a disproportionate amount of the antler hammer struck flakes were on tools used to work nettle stems. As nettle caused identification problems for all blind testers, its conflation with antler hammer struck flakes is a potentially distorting factor. If the nettle working retouch flakes are removed from the analysis, the difference in the proportion of correct determinations for flakes produced with different hammer types is reduced to 4%, suggesting that hammer type had relatively little effect on the correct identification of wear traces.

### The effect of flake size on accuracy

Analyses of dispersion (Fig 3) demonstrate similar distributions in the weight of incorrect responses for all three participants. Generally, and despite the contrasting number of incorrect responses between Blind Tester 2 and Blind Tester 1 and 3, incorrect responses in the majority of cases are on flakes that weigh less than 0.25g. Only in one instance was a flake weighing greater than 0.25g incorrectly identified (Flake 33, weight: 0.68g).

When examined through a GLM, statistical significance (in parameter values and to a 0.05 alpha level) between the weight of the artefact and the participant's response can be observed for the first and third participant (*z*: -2.861; *p*: 0.0042 and *z*: -2.008; *p*: 0.0446 respectively). For the second participant, statistical significance could not be observed between the two variables (*z*: -1.340, *p*: 0.180). With the limited sample size of incorrect responses for each participant, the statistical scores must be treated with some degree of caution.

**Fig 13:**
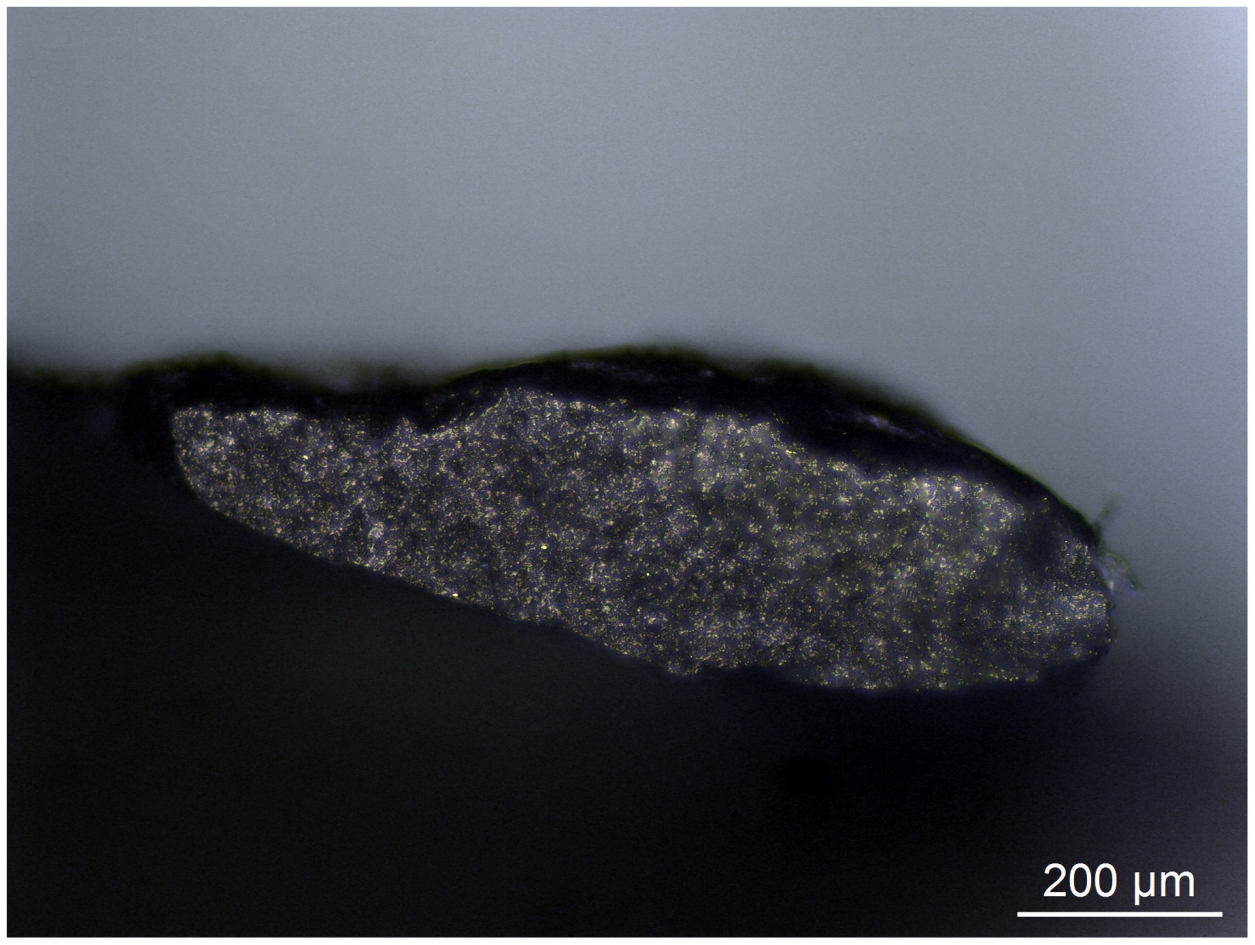
The butt of Flake 18, removed from a tool used to scrape dry hide. The external platform edge is on the upper side of the butt (100x).

These tests however suggest that for individuals where the proportion of accurate identifications are high, size is a significant factor in the limited number of incorrect identifications. Whereas, for individuals where the proportion of accurate identifications is lower there are a number of contributory factors leading to misidentification, meaning that size does not correlate so directly with inaccuracy. Moreover, the number of correct determinations on the smallest of retouch flakes by all blind testers indicates that size is not a singularly limiting factor in the correct identification of wear traces. For example, the smallest flake in the blind test was Flake 18, which weighed 0.0002g, with a butt which measured just 960 μm by 240 μm (Fig 13). Despite its size, Blind Tester 1 was able to identify that the flake came from a tool that had been used transversally to scrape an abrasive dry hide.

## Discussion

Feedback from the blind testers made it clear that the identification and interpretation of traces on the retouch flakes was difficult in comparison to studying complete tools. This was mainly due to the size of the retouch flakes, the limited areas of the traces that could be analysed, and the disassociation of the retouch flakes from the use-edges of the tools from which they had been struck. In this sense, studying wear traces without the broader context of the tool itself was a relatively unfamiliar and challenging experience for the blind testers. However, within the blind test the use-wear analysis of retouch flakes correctly revealed whether flakes were removed from used or unused edges on average nearly 85% of the time, with the directionality of use being correctly identified in 68% of cases.

The high proportion of correctly identified flakes for the used/unused category is by itself a validation of the application of use-wear analysis to retouch flakes, as it shows that, if present, it is possible to consistently identify resharpening flakes within an assemblage of microdebitage. This represents an important step forward in broadening the range of crucial questions that can be addressed by studying lithic microartefact assemblages.

Beyond this initial level of analysis, general categories of contact material were correctly identified in 53% of cases, and partially correctly identified in a further 10% of cases, whilst specific contact materials were correctly identified in 48% of cases, and partially correctly identified in 17% of cases. Whilst lower, these figures indicate that important insights can be made in terms of understanding the materials that flakes from resharpened tools had been used upon. As detailed above, this is important as the analysis of microartefact distributions offers the rare potential for identifying and distinguishing between different types of activity areas across a site. Therefore, for example, the results of the blind test suggest that it would be possible to distinguish bone, wood and hide working areas from each other with reasonable confidence.

The key question is how the results of the blind test compare with those of more standard lithic microwear blind tests. Evans [1] has compared the results of 19 blind tests calculating that the average accuracy rates for identifying contact materials across all tests was 49.5%, whereas identification of directionality was identified correctly in 68.7% of cases. No figures are given for the correct identification of used versus unused objects. If we compare these average figures we can see that the accuracy rates for the microdebitage blind test are very similar when compared to previous blind-tests, and in the case of some contact materials are actually higher. The accuracy rates for identifying the directionality of use are directly comparable with the current blind test achieving an overall rate of 68.1% correct responses. Accuracy rates for identifying contact materials in this study are higher if partially correct answers are taken into account (64.7% responses correct or partially correct), and comparable if only correct answers are considered (47.9%). In comparison to previous blind tests the accuracy of identifications is remarkable when the small size of the objects is taken into consideration.

**Fig 14:**
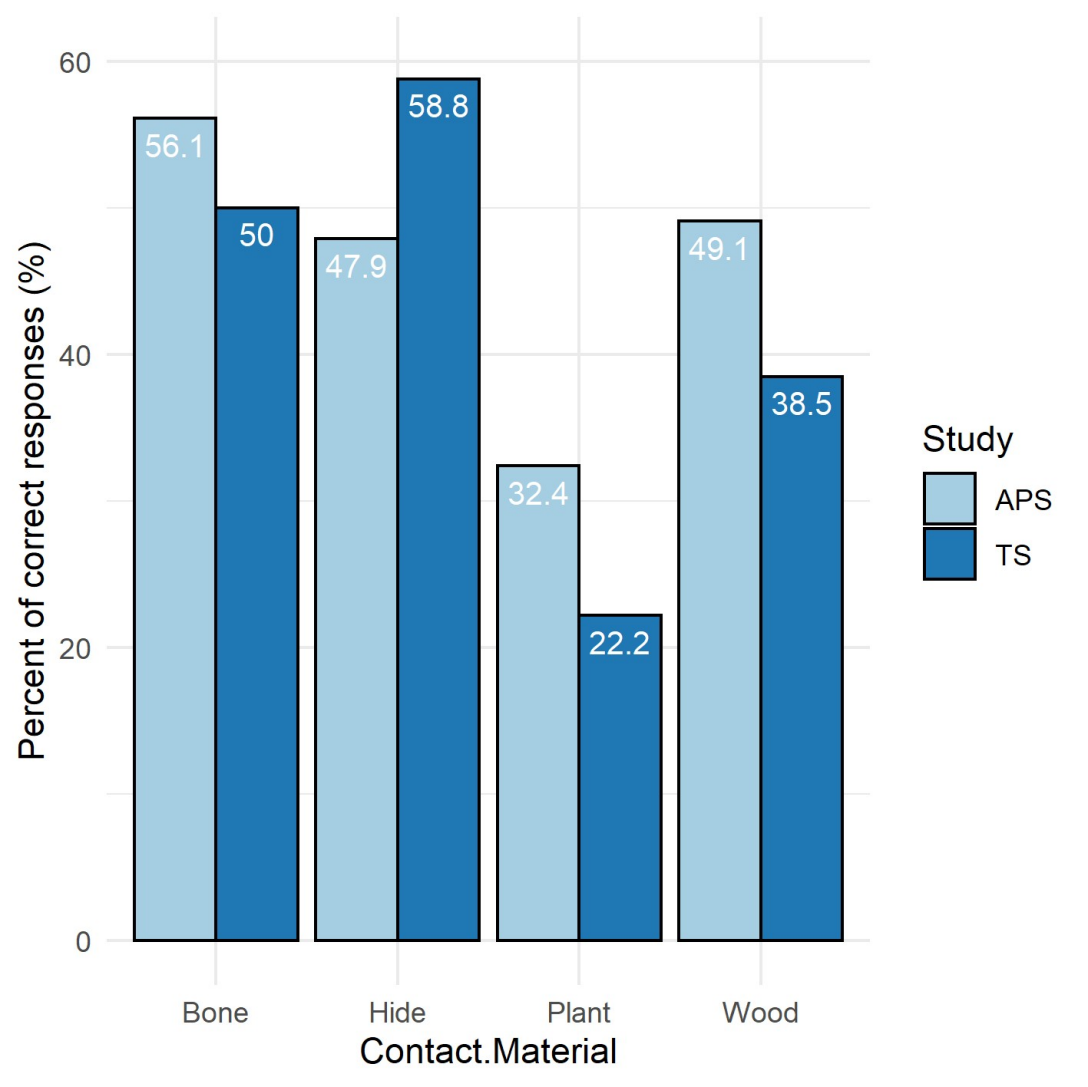
Comparison of the percentage of correct responses to the identification of different contact materials between all previous studies (APS) taken from Evans [1 Table 3] and this study (TS).

In addition to levels of overall accuracy, it is also useful to look at accuracy within the key contact materials of bone, hide, plant and wood. These data can also be compared with the collated data from previous blind tests compiled by Evans [1, Table 3]. The percentages of correct responses for each contact material in this study were compared against previous studies through a clustered bar chart (Fig 14). This highlights that, for three of the four contact materials (bone, plant and wood), the percentage of correct responses in this study (TS) were lower than all previous studies (APS), with a higher success percentage recorded in TS for hide. Despite this, general consistency in the results can be observed. This is supported by a Pearson's Chi-squared test for count data (*x*^*2*^: 9; *p*: 0.2133). The largest discrepancy between the two datasets is for the identification of wood. The reason is most probably that for the current blind test this category includes experiments involving stripping bark from green hazel branches, as well as working lime bark. The problems in identification that these materials caused has already been highlighted above and may relate to the fact that both materials produced traces that are characteristic of contact with materials softer than would be associated with standard woodworking traces. If the blind test data for wood for the current study was restricted to the pine working experiments the proportion of correct identifications rises to 47%, which compares closely to the collated dataset wood identification figure of 49.1% [1, Table 3]. Beyond this it can be seen that the lowest accuracy figures were for the identification of plant working, which was a particular issue within the current blind test but can also be seen to relate to a wider phenomenon across all blind tests. The data for directionality indicate that identifying the direction of tool use was also a problem for retouch flakes from tools used to work plants, particularly in cases where the direction of tool use was longitudinal.

In addition to the effect of contact material on identifying wear traces on retouch flakes, the results of the blind test have indicated that the type of hammer used to retouch the tool had little or no effect, whilst the size of flakes did have an affect but only to a minor degree. Indeed, the large variation in the level of accuracy between Blind Testers 1 and 3, when compared to Blind Testers 2, can certainly not be attributed solely to the size of the flakes. It is actually difficult to pinpoint any clear cut reasons for the disparity between the two groups of blind testers other than, as noted above, the fewer years of experience of Blind Tester 2.

**Fig 15:**
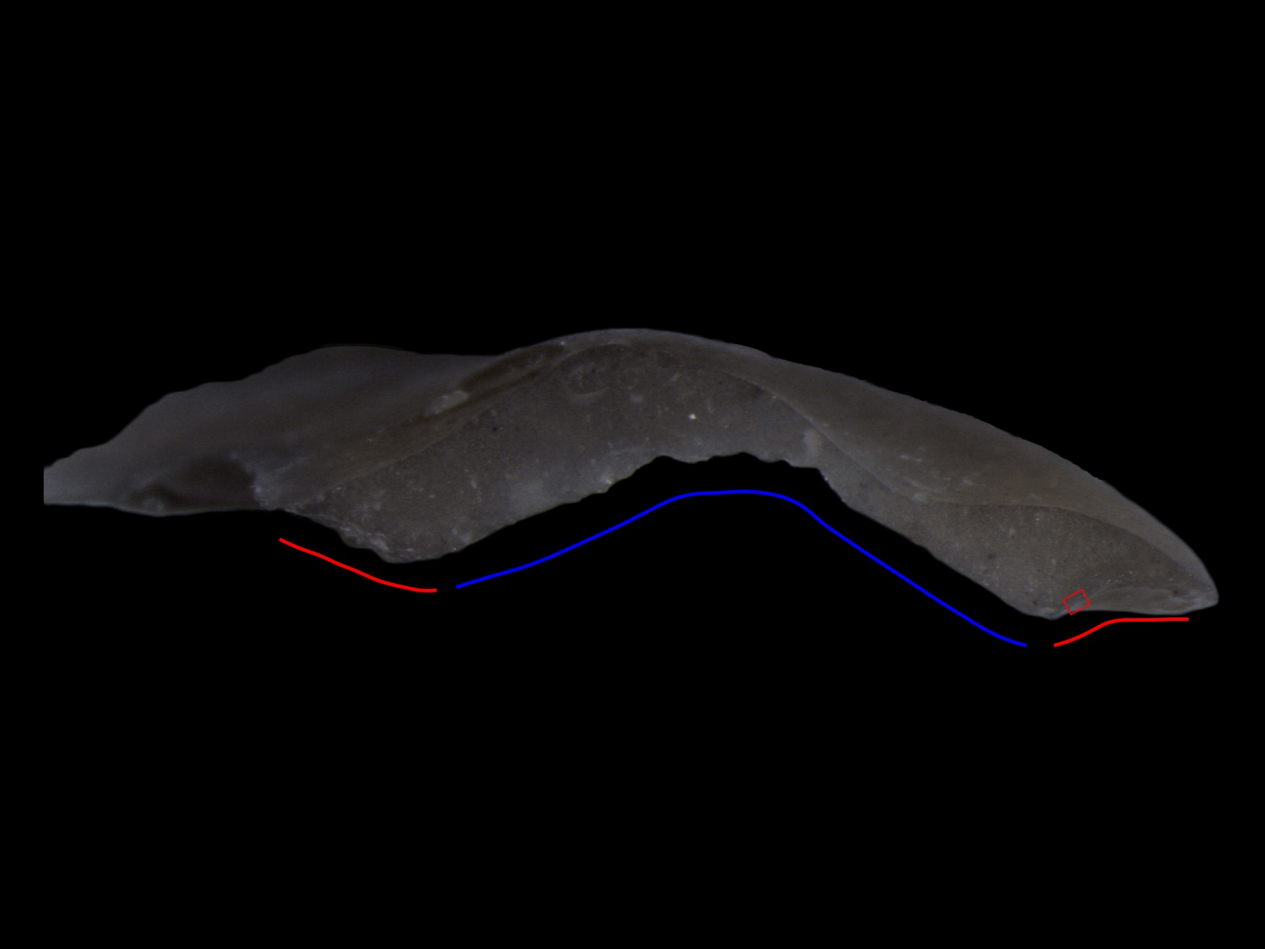
The butt of Flake 2, a retouch flake removed from a tool used to scrape fresh hide (12.5x). The external platform edge of the flake is on the lower edge of the butt. The part of the butt formed by the remnant of the original use-edge of the tool where wear traces are surviving is demarcated in red. The part of the butt where the use-edge has been removed by a previous retouch flake removal is demarcated in blue. The red box denotes the location of micrograph D in Fig 2.

In terms of the experience of conducting the experiments and blind test, there are a few salutary points that are worth noting. The wear traces on microdebitage can sometimes be highly localised, covering microscopically small areas. This is especially the case for flakes that have had their butts crushed during retouching, when traces may only survive in small patches on their dorsal surface. It is also the case where a retouch flake is not one of the first to be removed from a retouched edge effectively making it a “secondary” flake. For example, Flake 2 was struck from a used edge with a flake already having been removed from directly in front of the point of impact of the second removal. This led to a situation where the only surviving parts of the original use edge of the tool are at the two lateral extremities of the external platform edge (Fig 15). The small size of the flakes also presents practical issues with their analysis. Presenting the surface to be analysed at 90° to the microscope objective is important within any use-wear analysis using incident light microscopy, however, for microdebitage the small size of the analysable surface makes it absolutely paramount. Without doing so it is very easy to entirely miss wear traces. Therefore, it is advisable to position the flake on its mount under a stereo microscope so that one can see the angle of the butt in relation to its mount, ensuring that it is ultimately placed perpendicular to the microscope objective.

## Conclusion

The results of the blind test indicate that the identification of wear traces on retouch flakes, and by extension, on microdebitage in general, are identifiable at levels comparable to those reported in blind tests of complete artefacts. In this respect the use-wear analysis of assemblages of microdebitage should be viewed as another tool for interrogating lithic assemblages, one that is particularly suited to investigating the locations of activity areas. The technique should also be viewed as a means for expanding sample sizes in assemblages where tools and other utilised artefacts are present in small numbers. The issue of small sample sizes is a particular issue in context specific analyses, for example, where only material from intact occupation surfaces such as house floors is being analysed.

Use-wear analysis of microdebitage also represents logistical differences to standard use-wear approaches in that its application across a site requires systematic recovery of microdebitage with the most reliable method being bulk sampling for floatation, which needs to be followed by the laborious process of residue sorting. Work within the broader field of microartefact studies has shown that complex site formation processes affect the distribution of microdebitage, particularly on sites with compacted house floors. Therefore, the application of use-wear analysis on microartefact assemblages should be employed to answer specific research questions, and only after the site formation processes affecting the assemblage has been satisfactorily understood. If these conditions are met, the analytical potentials of the technique are clear. The analysis of microdebitage is ideally positioned to critically evaluate site function, to identify the location and character of activity areas, and therefore to examine assumptions about the relationship between tools and the locations of the activities that they represent. In this respect, it could be argued that traditional use-wear approaches have often uncritically assumed a direct relationship between the location of tools and the tasks that there were used for. The use-wear analysis of microdebitage provides a means for assessing that relationship, whilst also providing an additional method for identifying activity areas. This allows us to see how different activities were distributed across sites, for example providing insight into which activities took place in open vs. closed areas of settlements. Understanding the choreography of practice across archaeological sites allows us to investigate the manner in which material traditions were created, maintained and transformed. Such analysis should be seen as a fundamental building block in our understanding of past societies and social change.

## Supporting information

S1 Table The responses to the blind test of Blind Testers 1–3. (XLSX)
